# Targeting the *Plasmodium falciparum* IspE Enzyme

**DOI:** 10.1021/acsomega.4c06038

**Published:** 2024-10-25

**Authors:** Eleonora Diamanti, Annina M. Steinbach, Lais P. de Carvalho, Henni-Karoliina Ropponen, Antoine Lacour, Rawia Hamid, Sidra Eisa, Patricia Bravo, Spyridon Bousis, Boris Illarionov, Markus Fischer, Mostafa M. Hamed, Nina C. Bach, Matthias Rottmann, Jana Held, Matthias Witschel, Stephan A. Sieber, Anna K. H. Hirsch

**Affiliations:** † Helmholtz Institute for Pharmaceutical Research Saarland (HIPS)-Saarland University, Department of Pharmacy, Helmholtz Centre for Infection Research (HZI), Campus Building E8.1, 66123 Saarbrücken, Germany; ‡ Saarland University, Campus Building E8.1, 66123 Saarbrücken, Germany; § Center for Protein Assemblies, Technical University of Munich, Ernst-Otto-Fischer-Straße 8, 85748 Garching, Germany; ∥ Institute of Tropical Medicine, University of Tübingen, Wilhelmstraße 27, 72074 Tübingen, Germany; ⊥ Swiss Tropical and Public Health Institute, Kreuzstrasse 2, 4123 Allschwil, Switzerland; # Universität Basel Petersplatz 1, 4003 Basel, Switzerland; ∇ Hamburg School of Food Science, Institute of Food Chemistry, Grindelallee 117, 20146 Hamburg, Germany; ○ German Centre for Infection Research (DZIF), 38124 Braunschweig, Germany; ◆ BASF-SE Carl-Bosch-Strasse 38, 67056 Ludwigshafen, Germany

## Abstract

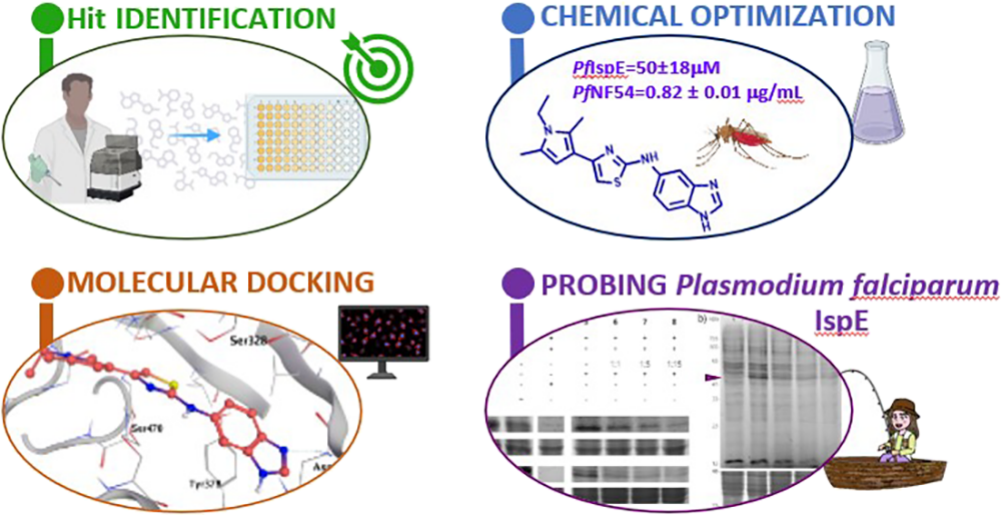

The enzyme IspE in *Plasmodium falciparum* is considered an attractive drug target, as it is essential for parasite survival and is absent in the human proteome. Yet it still has not been addressed by a small-molecule inhibitor. In this study, we conducted a high-throughput screening campaign against the *Pf*IspE enzyme. Our approach toward a *Pf*IspE inhibitor comprises *in vitro* screening, structure–activity relationship studies, examining the docking position using an AlphaFold model, and finally target verification through probe binding and sodium dodecyl sulfate-polyacrylamide gel electrophoresis (SDS-PAGE) analysis. The newly synthesized probe containing a diazirine and an alkyne moiety (**23**) allowed us to demonstrate its binding to IspE in the presence of a lysate of human cells (HEK293 cells) and to get evidence that both probe **23** and the best inhibitor of the series (**19**) compete for the same IspE binding site.

## Introduction

Isoprenoids (or terpenoids) are a large class of natural products that serve many essential biological functions in an organism.^1^ They are biosynthesized from the universal five-carbon building blocks isopentenyl diphosphate (IDP) and its isomer dimethylallyl diphosphate (DMADP).^2^ Eukaryotes, archaea, and a few bacteria obtain IDP and DMADP through the mevalonate pathway (MVA),^3^ while plants, algae, most eubacteria, and the Apicomplexa rely on the distinct methyl erythritol phosphate (MEP) pathway.^4^ The latter is absent in humans and found in the apicoplast of *Plasmodium falciparum*, the causative agent of malaria.^5^

**Scheme 1:**
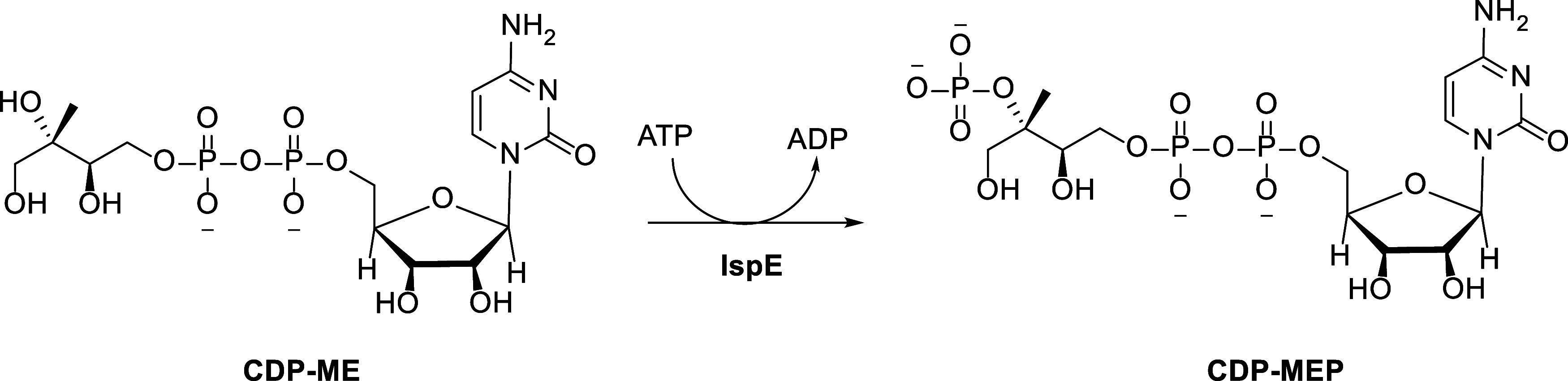
Fourth Step in the MEP Pathway The substrate 4-diphosphocytidyl-2*C*-methyl-d-erythritol (CDP-ME) is phosphorylated to 4-diphosphocytidyl-2*C*-methyl-d-erythritol-2-phosphate (CDP-MEP) by the kinase IspE.

The apoplast (apicomplexan plastid) is an indispensable organelle for the survival of the parasite, and it is highly likely that both IDP and DMADP are produced in the apicoplast, but the mechanism by which IDP is transported out of the plastids is poorly understood.^6^ As the MEP pathway is also essential in most Gram-negative bacteria, such as *Escherichia coli* or *Pseudomonas aeruginosa*, the enzymes of this pathway are considered promising anti-infective targets.^7^ However, owing to the hydrophilic substrates, the active sites of the enzymes of the MEP pathway are highly polar, making them particularly challenging drug targets. The synthesis of IDP and DMADP consists of eight steps that are catalyzed by seven enzymes. The focus of the current study is on the fourth enzyme of the pathway, the so-called 4-diphosphocytidyl-2*C*-methyl-d-erythritol kinase (IspE) enzyme (Scheme 1). It is the only adenosine triphosphate (ATP)-dependent enzyme in the MEP pathway, responsible for transferring the γ-phosphoryl group from ATP to the 2-OH group of 4-diphosphocytidyl-2*C*-methyl-d-erythritol (CDP-ME), yielding 4-diphosphocytidyl-2*C*-methyl-d-erythritol-2-phosphate (CDP-MEP).

**Figure 1:**
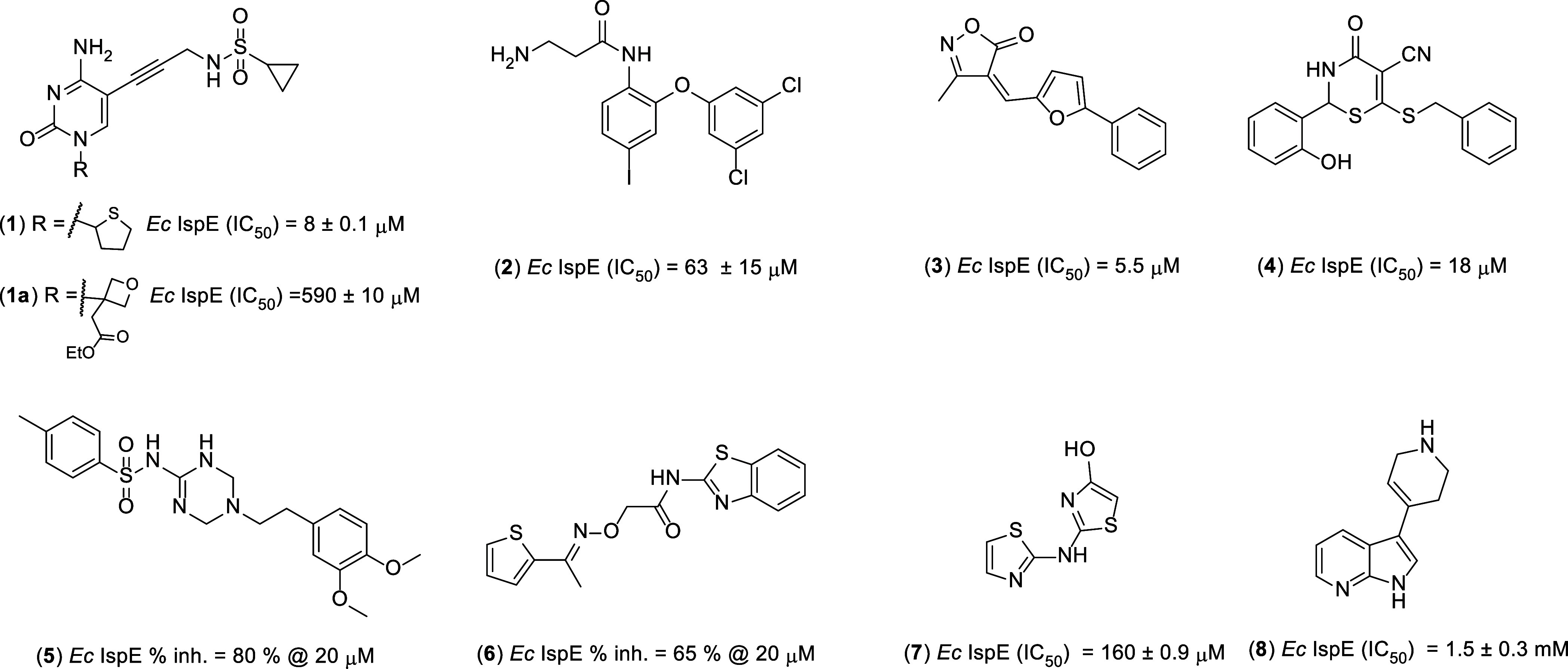
Chemical structures of reported IspE inhibitors.

IspE is a member of the galactose/homoserine/mevalonate/phosphomevalonate (GHMP) kinase superfamily with a two-domain architecture and an α/β-fold.^8^ The catalytic core is in a deep cleft at the interface of the cofactor and substrate-binding (CDP-ME) domains. Phylogenetic analysis revealed that the IspE enzymes are a distinct group from the human mevalonate kinase,^9^ and druggability assessment confirmed the presence of druggable pockets in the IspE enzyme.^10^ These findings encouraged us to search for novel IspE inhibitors. Further support is given by fosmidomycin, an inhibitor of 1-deoxy-d-xylulose-5-phosphate reductoisomerase (DXR or IspC), that is in clinical trials for the treatment of malaria.^11^ Nonetheless, no IspE inhibitor with concomitant cellular activity has been described so far. Over the years, the extensive work of our consortium on this enzyme enabled the identification of a single-digit micromolar *Ec*IspE inhibitor **1** and its more soluble analog **1a** that has been cocrystallized with the nonpathogenic *Aquifex aeolicus* IspE.^12^ Compound **1** (Figure 1) acts as a substrate (CDP-ME)-competitive inhibitor, filling the cytidine-binding pocket and an adjacent small hydrophobic subpocket. Recently, we explored the ATP-binding site and through virtual screening followed by a focused structure–activity relationship (SAR) study, we identified a novel IspE inhibitor **2** that does not belong to one of the known inhibitor classes.^13^ Other groups worked on the IspE enzyme and exploited other hit-identification approaches that afforded hits with promising inhibitory potency on the target but lacking whole-cell activity. Tang et al. followed two methodologies: they (i) tested a library of existing GHMP kinase inhibitors that led to compounds **3** and **4** (Figure 1) and (ii) performed an *in silico* screening of a library comprising two million compounds that produced hits **5** and **6**.^14^ The Brenk group performed virtual screening on the cytidine-binding moiety of *Aa*IspE followed by *in vitro* testing and found hits **7** and **8**.^15^ Furthermore, also, the exploitation of the target-directed dynamic combinatorial chemistry (tdDCC) as a hit-identification method using IspE from *Mycobacterium tuberculosis* (*Mtb*IspE) did not lead to any promising hits.^16^ Therefore, despite various hit-identification strategies and substantial efforts, a potent IspE inhibitor with whole-cell activity is still missing. Recently, we also experienced challenges and obstacles on the way to find a perfect balance between the on-target and whole-cell profile, in particular, when we aimed to target the enzymes of the MEP pathway.^13^ The situation is even more challenging if we look at the *P. falciparum* IspE enzyme where a crystal structure is missing. In fact, until now, the crystal structures of IspE orthologues that have been elucidated are for *A. aeolicus* IspE (*Aa*IspE, PDB ID: 2VF3), *E. coli* IspE (*Ec*IspE, PDB ID: 10J4),^8^*Thermus thermophilus* (*Tt*IspE, PDB ID: 1UEK),^17^ and *M. tuberculosis* (*Mtb*IspE, PDB ID: 3PYD),^18^ whereas for *P. falciparum* species, only a sequence analysis has been performed.^9^

To circumvent this challenge and increase our understanding of the target *Pf*IspE, we set out to find a *Pf*IspE inhibitor that can serve as a probe molecule and a prototype for future anti-infective drug discovery.

## Results and Discussion

### Hit Identification and Chemical Optimization

Here, we initiated a high-throughput screening (HTS) campaign on the *Pf*IspE protein (Section S1 in the Supporting Information). The HTS was based on a proprietary library of 100 000 compounds from the BASF company and used the inhibitory potency of the compound against the IspE protein as the readout. The IC_50_ from the IspE assay was also a key parameter for our chemical optimization study. Therefore, it is important to note that the activity of the target enzyme in the IspE assay is coupled to the oxidation of NADH (which is followed spectrophotometrically at 340 nm) via a cascade of the auxiliary enzymes pyruvate kinase and lactate dehydrogenase (PK/LDH, Section S2 in the Supporting Information). Accordingly, to confirm that the effects observed in the IspE assay are due to inhibition of the target enzyme rather than the auxiliary enzymes, it is crucial to maintain a difference between the PK/LDH and IspE enzymatic activities. Furthermore, as we have previously reported, the biological testing of this chemical class requires careful handling such as storage of the compounds at −20 °C.^19^ Based on what has been reported in the literature, a risk-mitigation strategy involves adding a substituent to the *C*-5 position of the thiazole ring,^20^ which we have adhered to in this SAR exploration.

**Figure 2:**
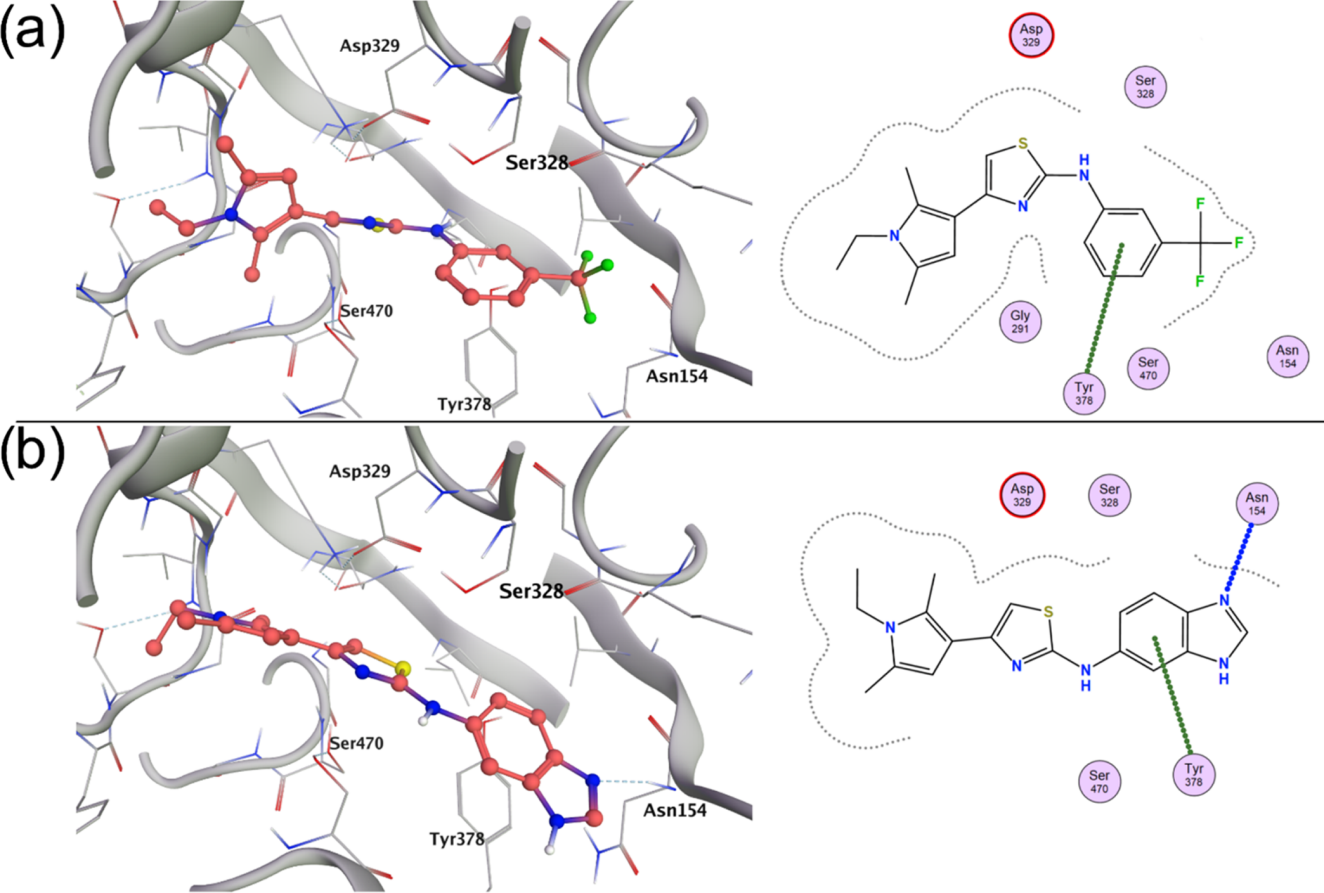
(a) Binding mode of **9** in the CDP-ME binding site of *Plasmodium falciparum* IspE. The phenyl ring is involved in a π–π interaction with the side chain of Tyr378. (b) Binding mode of **19** in the CDP-ME binding site of *P. falciparum* IspE. The benzimidazole nitrogen atom is involved in an H-bond interaction with the backbone NH of Asn154. The benzimidazole ring is involved in a π–π interaction with the side chain of Tyr378.

Despite the challenging nature of the target protein, this HTS campaign revealed hit compound **9** (IC_50_*Pf*IspE= 405 ± 114 μM) albeit with a moderate potency. However, this compound has promising features including (i) an aminothiazole ring that resembles the previously reported IspE inhibitor **7**; (ii) a good fit with the AlphaFold-predicted *Pf*IspE structure in the CDP-ME binding site via docking^21^ (Figure 2a and see Section S3 in the Supporting Information); and (iii) a modular structure that lends itself to chemical modification. As the next step, aiming to improve the inhibitory potency of **9** on the IspE enzyme, while keeping the substitution pattern of the pyrrole ring constant, we further explored the phenyl part.

Our experimental design comprised two steps: (1) we tested the sensitivity of the *ortho*- and *para*-positions of the trifluoromethyl group on the distal phenyl ring (Table 1, **9**–**11**) and (2) having identified the most favorable substitution pattern on the phenyl ring, we expanded the series of substituents varying the lipophilic, electronic, and steric properties (**12**–**20**). Synthetic procedures are reported in the Supporting Information, Sections S4 and S5. After comparing **9**–**11** and **12**, we disregarded further modifications at the *ortho*-position and compound **12** underlined the importance of having a substituted phenyl ring to achieve *Pf*IspE inhibition. Compounds **13** and **14** demonstrated that the introduction of an electron-donating group is detrimental to the inhibitory potency on the target, while a weak electron-withdrawing group such as chlorine in compound **15** seems to be favorable. Then, we continued on the chlorine series and synthesized **16** where the phenyl ring is replaced by a pyridyl and **17** bearing a bis-chlorine system. While both compounds are more potent than the initial hit **9**, these modifications did not lead to a better inhibition of IspE than **15**. Next, we introduced a primary amine as in **18** and bis-heterocycle system with endogenous nitrogen atoms, a benzimidazole **19** and an indazole ring **20**, respectively. Interestingly, these modifications led to a progressive increase in potency with benzimidazole derivative **19** as the best in the series, having an IC_50_ of 53 ± 19 μM against *Pf*IspE and no inhibitory potency toward the auxiliary enzyme (IC_50_ PK-LDH > 500 μM, Table 1).

**Table 1:**
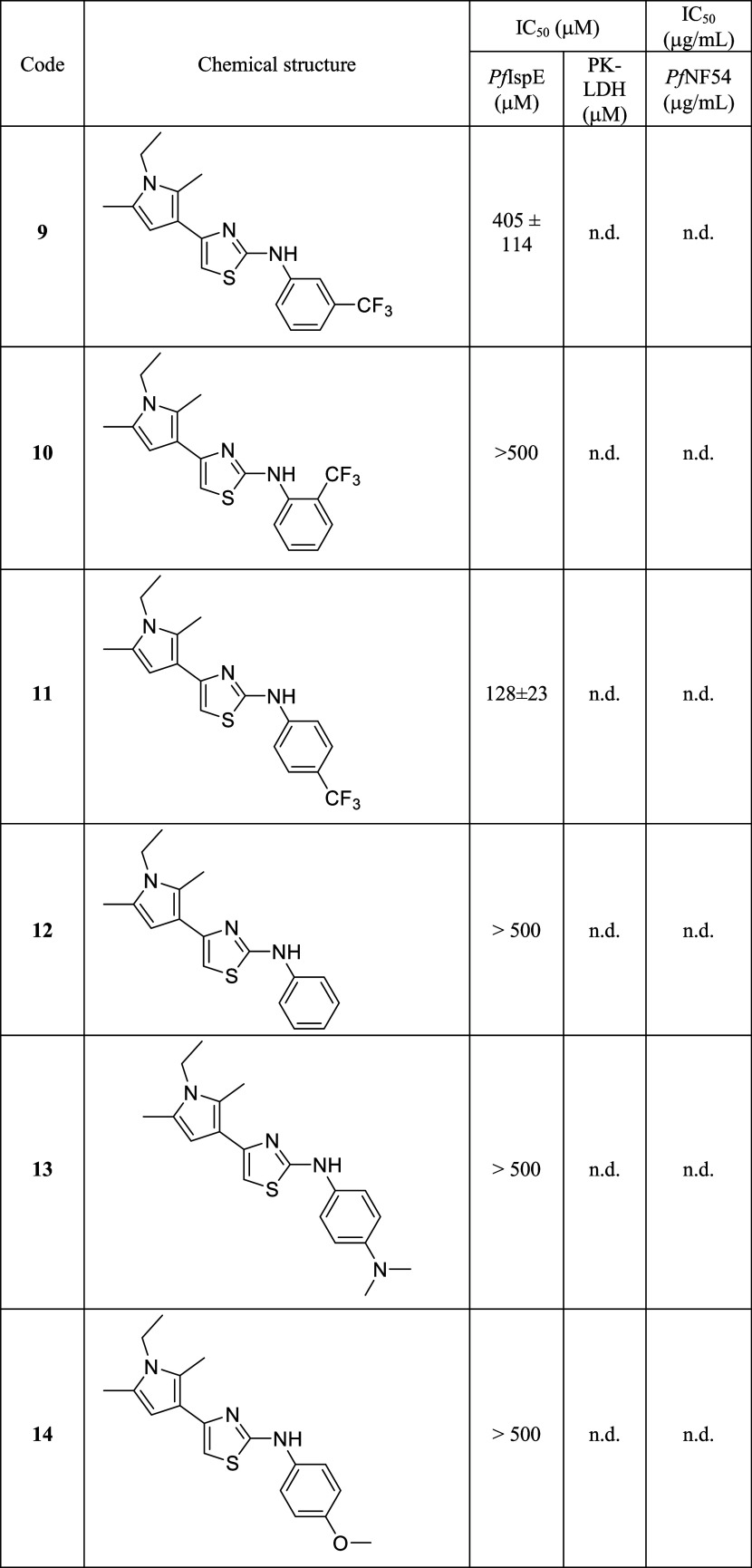

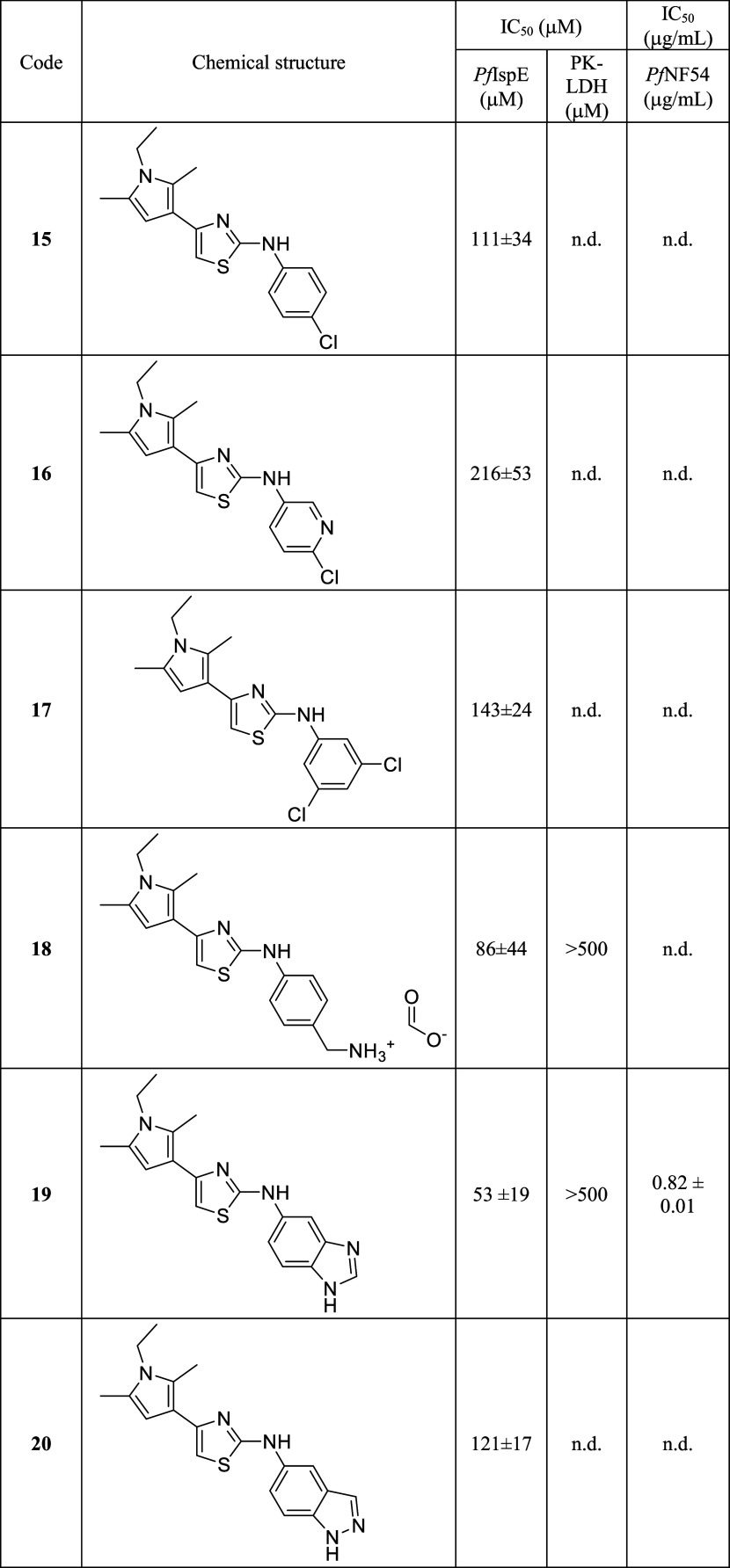

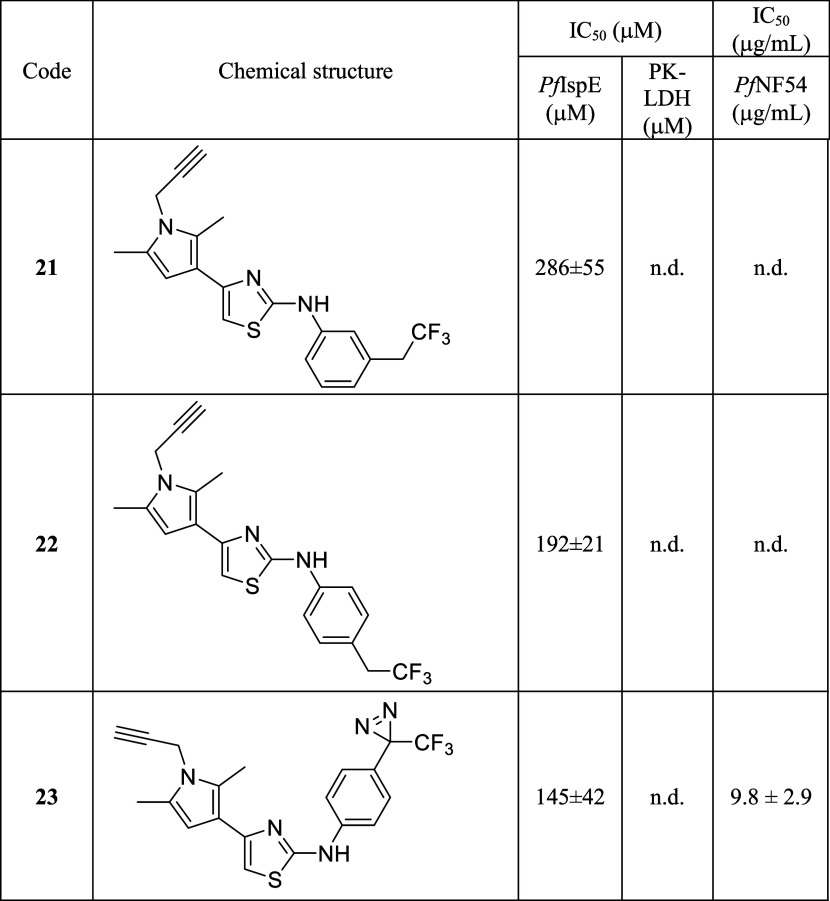
Inhibitory Potency on *Pf*IspE, PK-LDH, and *Pf*NF54 of Compounds **9**–**23**^a^

a *Pf*: *Plasmodium falciparum*; PK/LDH: pyruvate kinase/lactate dehydrogenase calculated for *Pf*IspE IC_50_ < 100 μM; n.d. = not determined.

As an inhibition against the target protein does not necessarily correspond to activity in the whole organism, we performed a [3*H*]-hypoxanthine assay (see Section S6 in the Supporting Information) to assess if compound **19** as the top-performing compound in the SAR study also inhibited the growth of *Pf*NF54 parasites. We found that parasite growth was inhibited with an IC_50_ of 0.82 ± 0.01 μg/mL (Table 1). This value (corresponding to ca. 2.4 μM) is lower than the IC_50_ for the isolated protein, which might appear surprising. However, direct comparison of the two assays is extremely difficult. Consequently, it is essential to note that the existence of additional target proteins within *P. falciparum* at this stage is neither confirmed nor ruled out. However, to the best of our knowledge, this result represents the identification of the first IspE inhibitor that is active also at the whole-cell level.

### Molecular Docking

To rationalize this 8-fold boost in IspE inhibitory potency compared to that of the initial hit **9**, we also performed molecular docking (MOE v2020) of both compounds in the CDP-ME binding site of the AlphaFold-predicted structure of *Pf*IspE. The binding site is characterized by the presence of several important residues. Indeed, Tyr378 occupies the same position as Phe185 in the *Ec*IspE structure (PDB: 1OJ4) and is hypothesized to be involved in the π–π stacking interaction with CDP-ME as this is the case in the *Ec*IspE structure. This residue is also involved in π–π stacking in the binding mode of analogs of compound **1** to *A. aeolicus* IspE (Tyr178, PDB: 2VF3).^12,200^ Additionally, the methyl-d-erythritol moiety is involved in hydrogen-bonding interactions with Asp141 and Lys10 in the *Ec*IspE structure, which equate to Asp329 and Lys139 in the AlphaFold-predicted structure of *Pf*IspE, respectively. One key difference between the CDP-ME binding sites of the two structures is the binding of the cytidine ring. Indeed, in *Ec*IspE, the cytidyl ring binds to the receptor through hydrogen bonds with His26. An equivalent His residue is not present in the AlphaFold-predicted structure of *Pf*IspE. We hypothesized that Asn154, which occupies the same space as His26 when aligning the structures, is instead involved in CDP-ME binding in *Pf*IspE; however, this has yet to be confirmed experimentally. The binding poses of compounds **9** and **19** are shown in Figure 2. Compound **19** forms a strong H-bonding interaction between the benzimidazole nitrogen atom and the backbone NH of Asn154 in addition to the π–π interaction with the side chain of Tyr378, which is also seen in the predicted binding mode of compound **9**. This additional interaction may explain the observed boost in the biochemical inhibitory potency of compound **19** relative to **9** through tighter binding and a better ability to displace CDP-ME from the binding site.

### Verification of IspE Binding

**Figure 3:**
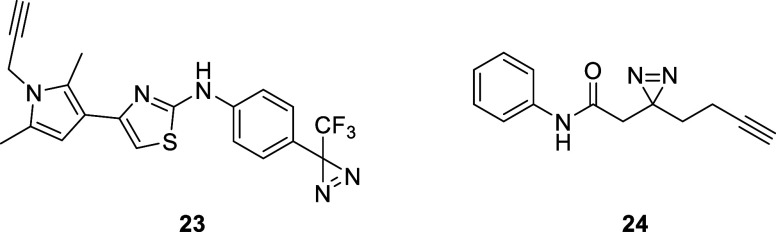
Chemical structure of photo-crosslinkers **23** and **24**.

To verify binding between our optimized compound and the IspE enzyme as the target protein in the two isoforms of the clinically relevant microorganisms *P. falciparum* and *E. coli*, we performed an affinity study. For this assessment of the predicted mode of action, we adopted an affinity-based proteome profiling (AfBPP) strategy.^22,23^ The first step in this approach was the design of an active probe, which had to contain two key elements: (i) a reactive group for covalent binding to the protein and (ii) a reporter tag for the detection of the labeled protein(s) from proteomes. Therefore, supported by the SAR study described above, we designed and synthesized two model chemical probes, **21** and **22**; synthetic procedures are reported in the Supporting Information, Sections S4 and S5. In agreement with our SAR, the *para*-substitution of the model probe is more favorable than the *meta*-substitution, prompting us to synthesize the corresponding diazirine probe **23** (Figure 3) to enable photochemical binding to proteins. Interestingly, we found that **22** and **23** are able to inhibit not only the *Pf*IspE enzyme but also the *E. coli* orthologue (Tables 1 and S1, Section S2 in the Supporting Information). In addition, **23** was also submitted to the whole-cell assay and exhibited an IC_50_ value of 9.8 ± 2.9 μg/mL against *Pf*NF54 cells. Both values are higher than those for compound **19** but still indicate a reasonably preserved activity as an inhibitor in the biochemical and whole-cell assays.

As a control for the following affinity experiments, we used our previously published compound **24**, a minimal aromatic photoprobe.^24^ This photoprobe only contains a simple aromatic group in addition to the photoreactive diazirine group, and the alkyne handle covalently links the reporter. It thus presents to potential binding partners only the functional groups introduced to convert compound **19** to photoprobe **23**. Hence, probe **24** can be used to detect specific binding to the photo-crosslinker moieties in contrast to the supposedly active parts of probe **23**.

**Figure 4:**
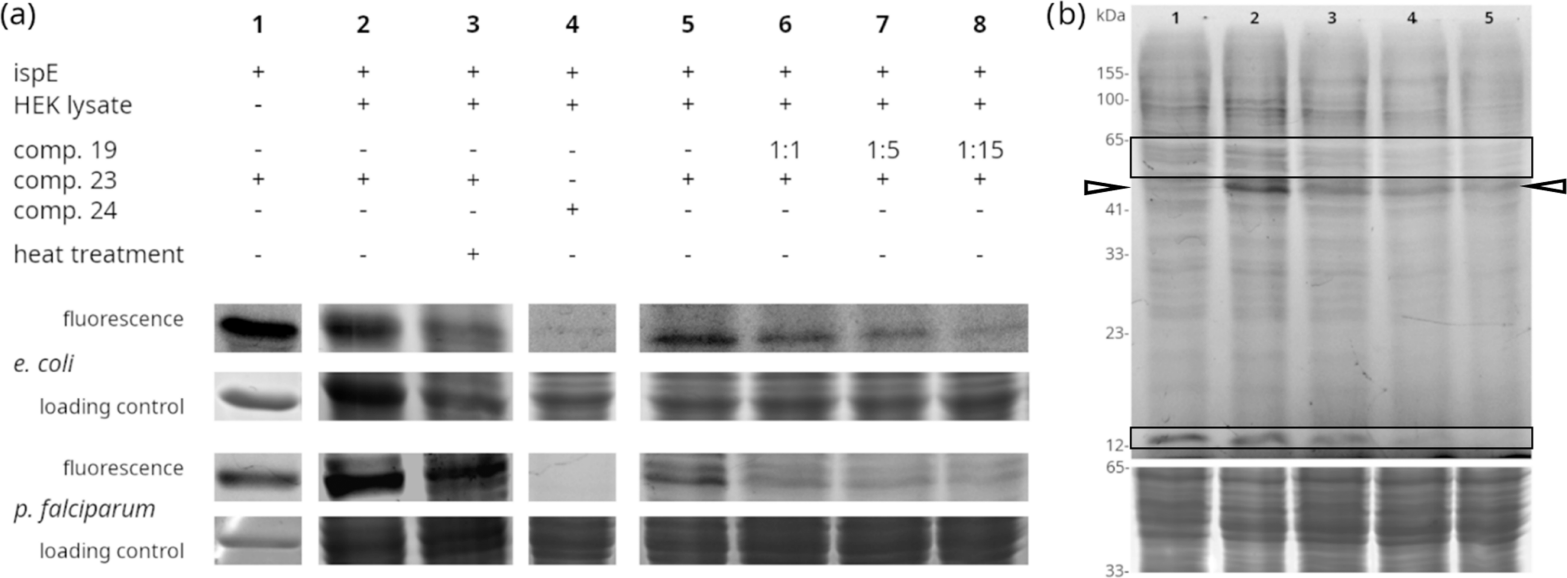
Verification of probe **23** binding to IspE proteins of *E. coli* and *P. falciparum*. (a) Analytical SDS-PAGE experiments with column 1 verifying the affinity of the probe for the target proteins, columns 2 and 3 comparing binding to the native and denatured protein with additional human HEK cell lysate as a background, column 4 testing IspE affinity to the minimal aromatic photo-crosslinker **24** (chemical structure shown in figure), columns 5–8: comparison of probe **23** binding in the presence of competing active compound **19**. The molar ratios of **23**:**19** are given above the respective lanes. (b) Fluorescence scan of a competition SDS-PAGE with HEK cell lysate without (lane 1) and with being spiked in *Pf*IspE protein (lanes 2–5). Dark bands correspond to areas with a prevalent affinity for probe **23**. In lanes 3–5, competitor **19** is present in increasing excess compared to the probe (lane 3:1:1, lane 4:1:5, lane 5:1:15). Below, the corresponding loading control is shown. *Pf*IspE (UniProt entry A0A1B1TK84) is expected to run at a molecular weight of 63 kDa, with *Ec*IspE (UniProt entry P62615) expected to run at 31 kDa. All depicted gel scans are included in their entirety in the Supporting Information, Section S7.6.

We used both the isolated *Pf* and *Ec*IspE proteins (Section S1 in the Supporting Information) to confirm their binding to probe **23** through sodium dodecyl sulfate-polyacrylamide gel electrophoresis (SDS-PAGE) (Figure 4a, column 1). When offering additional potential binding partners to probe **23** in the form of human cell HEK293 lysate as a background in addition to being spiked in *Pf*IspE and *Ec*IspE proteins, binding was slightly reduced but still pronounced (Figure 4a, column 2b, lane 2 marked by the triangles). We chose a human cell line to immediately assess if our chemical class interferes with the human off-target(s). The binding profile of **23** to the pure human lysate suggests the presence of such off-targets (Figure 4b, lane 1) with binding seeming more prevalent for some protein bands than for others, e.g., areas marked with boxes. When the combined lysate and spiked-in IspE proteins are subjected to heat denaturation prior to binding the probe (Figure 4a), the binding is lower and the bands are less defined, indicating dependence on the native structure.

As an additional control for unspecific binding, we verified that the aromatic minimal probe **24** (Figure 3) does not show the same binding profile.^24^ The fact that **24** was not sufficient to exhibit the same protein bands as **23** (Figure 4a, column 4, and Supporting Information, Figures S2–S5) further supports that the detected binding is mediated by the designed active region that compounds **23** and **19** have in common. When active compound **19** is present in addition to probe **23**, the fluorescence of the reporter group is reduced already at a competition ratio of 1:1 of **23**:**19** (Figure 4a, comp. columns 5 and 6), indicating that both compounds compete for the same binding site. The effect becomes progressively more pronounced with increasing concentration of **19** as shown in the series of bands in Figure 4a, columns 5–8, and Figure 4b, lanes 2–5, with the triangles marking the position of the *Pf*IspE bands. The relative fluorescence is reduced to about 37% for *EcIspE* and 52% for *Pf*IspE when compound **19** is present in 15 times excess compared to probe **23** (Figure S8). The competition between compounds **19** and **23** seems also to take place for some of the background binding to human proteins (Figure 4b, lanes 2 and 5, areas marked by boxes). This competition indicates that both compounds target the same binding sites in the proteins.

In summary, the binding experiments were able to demonstrate that probe **23** has a high affinity for the target protein IspE from both *P. falciparum* and *E. coli* in its native conformation. Furthermore, it seems to bind via the designed active regions, in contrast to photoprobe-specific moieties, and competes with compound **19** for the same IspE binding sites in both tested organisms.

## Conclusions

In the present study, we identified and optimized an inhibitor of the IspE enzyme in order to disrupt the MEP pathway, which is essential for the survival of our target pathogens *P. falciparum* and *E. coli* but absent in humans. The most promising compound derived from an initial HTS against IspE activity was the starting point for an SAR study that yielded compound **19** with a significantly increased inhibitory potency. Our final compound has an IC_50_ of 53 ± 19 μM and is thus the first inhibitor of *Pf*IspE exhibiting potency in the micromolar range. Although this value is still in the double-digit micromolar range, it paves the way for further work around this chemical class. Furthermore, the inhibitory potency of **19** in a whole-cell environment (*Pf*NF54, IC_50_ = 0.82 ± 0.01 μg/mL), reinforces its potential as a frontrunner chemotype. Biochemical affinity experiments confirmed direct IspE engagement of the compound in an *in vitro* environment. Moreover, we present the first *Pf*IspE AlphaFold model that exhibits a good correlation between the inhibitory activities and docking poses of our molecules.

In summary, this study is a promising starting point for investigating IspE and the MEP pathway in more detail, using our newly identified probe, and for further developing our prototype compound into an anti-infective drug targeting IspE to combat infections.

## Supporting Information Available

^1^H NMR, ^13^C NMR, and ^19^F NMR spectra, HPLC-MS, biological protocols, IspE and *Pf*NF54 inhibition assays, *in silico* studies, and AfBPP experiments (PDF)
