# Supplementary material for: Targeting the Plasmodium falciparum IspE Enzyme
Source: ACS Omega. 2024 Oct 25;9(44):44465–73. doi: 10.1021/acsomega.4c06038 (PMC11541488; doi:10.1021/acsomega.4c06038)
Supplement: Supplementary file 1 — ao4c06038_si_001.pdf [file ao4c06038_si_001.pdf]

Supporting Information

Targeting the *Plasmodium falciparum* IspE enzyme

Eleonora Diamanti[a‡#], Annina M. Steinbach[‡c], Lais P. de Carvalho[d], Henni-Karoliina Ropponen[a,b¥], Antoine Lacour[a,b], Rawia Hamid[a,b], Sidra Eisa[a,b], Patricia Bravo[e,f], Spyridon Bousis[a], Boris Illarionov[g], Markus Fischer[g], Mostafa M. Hamed[a], Nina C. Bach[c], Matthias Rottmann[e,f], Jana Held[d,h], Matthias Witschel[i], Stephan A. Sieber[a,c], and Anna K. H. Hirsch [a,b*]

[a]Helmholtz Institute for Pharmaceutical Research Saarland (HIPS) – Helmholtz Centre for Infection Research (HZI) Campus Building E8.1, 66123 Saarbrücken, Germany.

[b]Saarland University, Campus Building E8.1, 66123 Saarbrücken, Germany.

[c]Center for Protein Assemblies, Technical University of Munich, Ernst-Otto-Fischer-Straße 8, 85748 Garching, Germany.

[d]Institute of Tropical Medicine, University of Tübingen, Wilhelmstraße 27, 72074, Tübingen, Germany.

[e]Swiss Tropical and Public Health Institute, Kreuzstrasse 2, 4123 Allschwil, Switzerland.

[f]Universität Basel Petersplatz 1, 4003 Basel, Switzerland.

[g]Hamburg School of Food Science, Institute of Food Chemistry, Grindelallee 117, 20146 Hamburg, Germany.

[h]Deutschen Zentrum für Infektionsforschung (DZIF)

[i]BASF-SE Carl-Bosch-Strasse 38, 67056 Ludwigshafen, Germany.

‡E.D. and A.M.S. contributed equally to this paper.

*Email: anna.hirsch@helmholtz-hzi.de

Table of Contents

1.0 Isolation of *Plasmodium falciparum* IspE 3

1.1 Expression and Purification of *Pf*IspE 3

1.2. Protein Purification........ 4

2.0 Biochemical IspE assay 4

3.0 Docking analysis 6

3.1 Preparation of ligands 6

3.2 Preparation of protein structures 6

3.3 Structural modeling........ 7

3.4 Docking........ 7

4.0 General information........ 7

4.1 Chemicals, Materials and Methods........ 7

4.2 Abbreviations 8

5.0 Synthesis and characterization of compounds 9–23........ 8

5.1 Synthetic schemes........ 8

5.2 General procedures........ 11

5.3 Synthesis and Characterization of compounds 9─23........ 11

6.0 *In vitro* drug sensitivity assay on *Plasmodium falciparum* asexual blood stage parasites........ 21

7.0 Analytical SDS-PAGE........ 22

7.1 Background lysate 22

7.2 Reaction with probe 23 22

7.3 Click reaction 23

7.4 SDS-PAGE........ 23

7.5 Post-processing 24

7.6 Complete SDS-PAGE scans 24

Figure S2: Scan of Coomassie stained gel. 25

Figure S3: Fluorescence scan of the gel in Figure S2........ 25

Figure S4: Scan of Coomassie stained gel. 26

Figure S5. Fluorescence scan of the gel in Figure S4........ 26

Figure S6. Scan of Coomassie stained gel........ 27

Figure S7. Fluorescence scan of the gel in Figure S5........ 27

7.7 Quantification of relative fluorescence in competition experiments........ 28

Figure S8. Relative normalized fluorescence in competition series with EcIspE and PfIspE........ 28

Spectra of some representative compounds 29

References.. 37

1.0 Isolation of *Plasmodium falciparum* IspE

1.1 Expression and Purification of *Pf*IspE

The pET22- *Pf*IspE- plasmid was transformed into competent *Escherichia coli* BL21 (DE3) Arctic cells (Agilent) using standard heat shock protocol. Transformed cells were plated onto Luria-Bertani (LB) agar supplemented with ampicillin at a concentration of 50 mg/mL.

A single colony of transformed cells was used to inoculate 50 mL of 1.5XLB medium containing ampicillin (50 μg/mL). The preculture was incubated overnight at 30 °C with shaking at 200 rpm. From the preculture, an appropriate volume (dilution factor 1 to 100) was transferred to 500 mL of fresh 1.5x LB medium with ampicillin and incubated at 30 °C with shaking until the optical density at 600 nm (OD600) reached approximately 0.3. The culture was then transferred to a shaker set at 16 °C for 45 minutes, and IPTG was added to a final concentration of 120 mg/L. The culture was further incubated at 16 °C for 2 days.

Cells were harvested by centrifugation at 8000 rpm for 15 minutes at 4 °C. The cell pellet was resuspended in cold saline solution (0.9% NaCl) at a volume of approximately 5 mL per gram of cell paste. After resuspension, cells were centrifuged again under the same conditions. The resulting cell pellet was stored at -80 °C until further use.

For cell lysis, the frozen cell pellet was thawed on ice and resuspended in lysis buffer containing 50 mM Tris-HCl pH 8.0, 400 mM NaCl, and 0.02% NaN_3, supplemented with 15 mM imidazole at a volume of approximately 4 mL per gram of cell paste. The cells were disrupted by passing through a French-Press twice at a pressure of 1.8

kBar. The lysate was then centrifuged at 15000 rpm for 80 minutes at 4 °C to remove cell debris, and the supernatant was collected.

1.2. Protein Purification

The supernatant containing the target protein was applied to a Ni-chelating Sepharose column (1 cm x 20 cm) that had been equilibrated with the lysis buffer containing 15 mM imidazole. The column was washed with the same buffer supplemented with 15 mM imidazole until the OD280 reached the baseline. The bound proteins were then eluted from the column using a gradient of imidazole ranging from 15 mM to 600 mM, collected in a volume of 100 mL.

If further purification was required, size exclusion chromatography was performed using a Superdex200 (GE, 2 cm x 60 cm) column. The column was equilibrated with the lysis buffer without imidazole. The eluted IspE fractions from the previous chromatography step were directly applied to the top of the Superdex200 column.

The purity and integrity of the purified *Pf*IspE protein were assessed using SDS-PAGE. The molecular weight of the protein was estimated by comparison with standard protein markers. The concentration of the purified protein was determined using a NanoDrop. The purified *Pf*IspE protein was aliquoted and stored at - 80 °C until further use.

(Note: All procedures were performed at 4 °C unless otherwise specified).

2.0 Biochemical IspE assay

Escherichia coli IspE[1] and *Plasmodium falciparum* IspE[2] for IspE assays was expressed, isolated and purified as previously described. All other enzymes and chemicals used in the assays were purchased from Sigma-Aldrich (Taufkirchen, Germany).

For testing of compounds in the IspE assay, CDP-ME (0.2 mM) in 100 mM Tris-HCl, pH 7.6, 0.02% NaN_3 (30 µL) was added to a well of the 384-well microplate, preloaded either with DMSO or with test compound dissolved in DMSO (3 µL). The reaction was started by addition of 100 mM Tris-HCl, pH 7.6, 10 mM $MgCl_2$, 60 mM KCl, 10 mM dithiothreitol, 0.02% NaN_3, 1 mM NADH, 2 mM phosphoenolpyruvate, 2 mM ATP,

pyruvate kinase (1 U mL^{-1}), lactate dehydrogenase (1 U mL^{-1}) and *E. coli* IspE (0.05 U mL^{-1}) (27 µL per microplate well).

IC_{50} values were measured at CDP-ME concentration 100 µM.

In order to find out if tested compounds are active *vs* auxiliary enzymes from the IspE assay, they were additionally tested in the pyruvate kinase assay. For this purpose, 1 mM ADP in 100 mM Tris-HCl, pH 7.6 (30 µL) was added to a well of the 384-well microplate which had been preloaded with DMSO or with test compound solved in DMSO (3 µL). The reaction was started by addition of 100 mM Tris-HCl, pH 7.6, 10 mM $MgCl_2$, 60 µM mM KCl, 10 mM dithiothreitol, 0.02% NaN_3, 1 mM NADH, 2 mM phosphoenolpyruvate, pyruvate kinase (0.05 U mL^{-1}) and lactate dehydrogenase (0.05 U mL^{-1}) (27 µL per microplate well).

For both kinds of assays the OD values in the microplate wells were monitored photometrically at 340 nm (room temperature) for 30 to 90 min in a plate reader (SpectraMax5, Molecular Dynamics, USA). Initial rate values were evaluated with a nonlinear regression method using the program Dynafit.[3]

Figure S1 : IC_{50} curve of compound **19**.

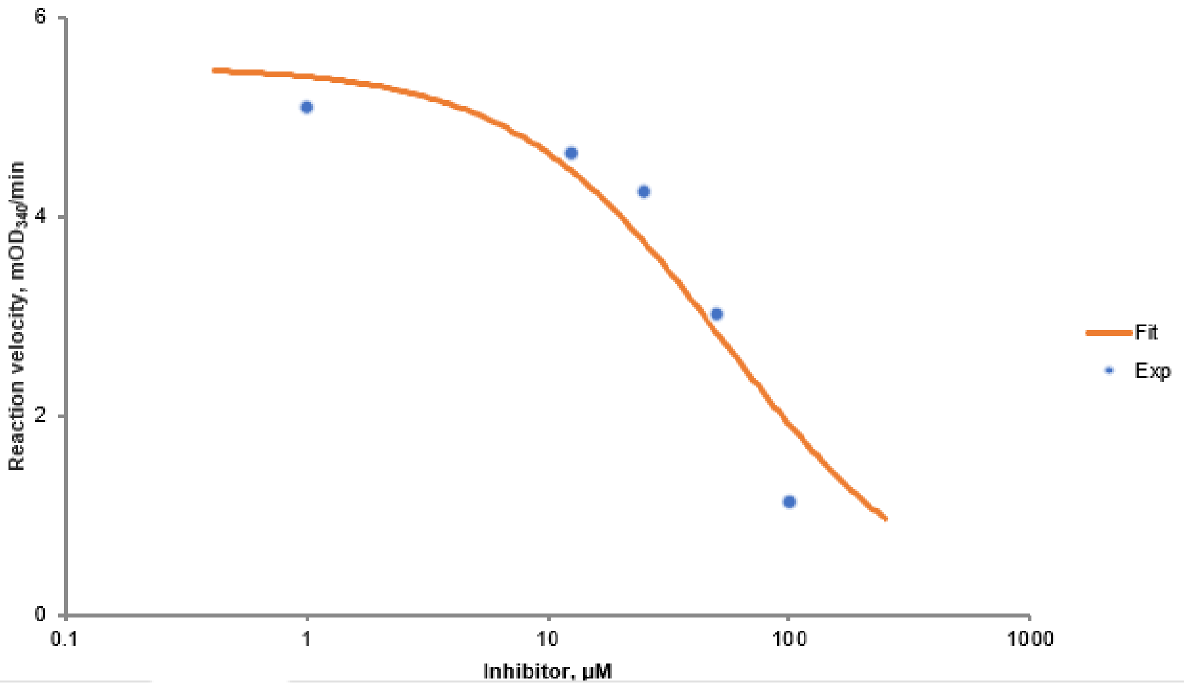

*Pf*IspE IC_{50}= 53 ±19 µM for substrate concentration of 100 µM

Table S1: Inhibitory potency on *Ec*IspE.

Code	*Ec*IspE (μM)
**21**	>500
**22**	31±13
**23**	77±16

3.0 Docking analysis

MOE v2020.09 was used for the subsequent analysis.

3.1 Preparation of ligands

The 2D structures of Compounds **9** and **19** were sketched using ChemDraw professional 20.0 and were imported into the MOE window. The compounds were subjected to an energy minimization up to a gradient of 0.001 kcal mol^{-1} $Å^2$ using the MMFF94x force field and R-field solvation model, then they were saved as an .mdb file. The predominant protonation status of the compounds in aqueous medium at pH 7 was calculated via the compute **|** molecule **|** wash command in the database viewer window.

3.2 Preparation of protein structures

The Alphafold predicted structure of *P. falciparum* IspE was used in the docking experiments. The structure was downloaded from the UniProtKB website using accession code A0A1B1TK84. The pdb file was then loaded into the MOE interface using default settings. The potential was set up to Amber10:EHT as a force field and R-field for solvation. After removal of the co-crystalized ligand, addition of hydrogen atoms, removal of water molecules farther than 4.5 Å from ligand or receptor, correction of library errors were performed via the QuickPrep module.

3.3 Structural modeling

The binding site residues were selected based on available homologous crystal structures where the substrate CDP-ME is bound (PDB ID: 2V8P from *Aquifex aeolicus* IspE).

3.4 Docking

Docking was performed for compounds **9** and **19** in the CDP-ME binding site. In all cases, placement trials were set to 100 poses with triangle matching placement and rigid receptor refinement. The refinement scoring function was GBVI/WSA dG with 10 poses.

4.0 General information

4.1 Chemicals, Materials and Methods

NMR experiments were run on a Bruker Ultrashield plus 500 (500 MHz) spectrometer. Spectra were acquired at 300 K, using deuterated dimethylsulfoxide (DMSO−d_6) as solvent. Chemical shifts for ^{1}H and ^{13}C spectra were recorded in parts per million (ppm) using the residual non-deuterated solvent as the internal standard (for DMSO−d_6: 2.50 ppm, ^{1}H; 39.52 ppm, ^{13}C). Coupling constants (J) are given in Hertz (Hz). Data are reported as follows: chemical shift, multiplicity (s = singlet, d =doublet, t = triplet, m = multiplet, br = broad and combinations of these) coupling constants and integration. Flash chromatography was performed using the automated flash chromatography system CombiFlash Rf+ (Teledyne Isco, Lincoln, NE, USA) equipped with RediSepRf silica columns (Axel Semrau, Sprockhövel Germany). TLC was performed with aluminium-backed silica TLC plates (Macherey-Nagel MN ALUGRAM Sheets SIL G/UV 254 20 x 20cm 818133) with a suitable solvent system and was visualized using UV fluorescence (254 & 365 nm).

Mass spectrometry was performed on a Dionex UltiMate 3000 -MSQ LCMS system (Thermo Fisher, Dreieich, Germany) using a Hypersil Gold column, 150 x 3 mm, 5 µm. At a flow rate of 700 µL/min, the gradient of H_2O (0.1% FA) and ACN (0.1% FA) starting

from 30% ACN and then increased to 95% over 12 min. The mass spectrum was measured in positive and negative modes in a range from 100–600 *m/z*. The UV spectrum was recorded at 254 nm. High-resolution mass spectra (HR-MS) were recorded with a ThermoScientific system where a Dionex Ultimate 3000 RSLC was coupled to a Q Exactive Focus mass spectrometer with an electrospray ion (ESI) source. An Acquity UPLC BEH C8, 150 x 2.1 mm, 1.7 µm column equipped with a VanGuard Pre-Column BEH C8, 5 x 2.1 mm, 1.7 µm (Waters, Germany) was used for separation. At a flow rate of 250 µL/min, the gradient of (A) H_2O + 0.1% FA and (B) ACN + 0.1% FA was held at 10% B for 1 min and then increased to 95% B over 4 min. It was held there for 1.2 min before the gradient was decreased to 10% B over 0.3 min where it was held for 1 min. The mass spectrum was measured in positive mode in a range from 120–1000 m/z. UV spectrum was recorded at 254 nm.

4.2 Abbreviations

Dichloromethane (DCM), ethyl acetate (EtOAc), ethanol (EtOH), formic acid (FA), hydrochloric acid (HCl), dipalladium-tris(dibenzylideneacetone)chloroform complex ($Pd_2(dba)_3 \cdot CHCl_3$), sodium sulfate (Na_2SO_4), sodium hydroxide (NaOH), tetrahydrofuran (THF), trifluoroacetic acid (TFA). Other abbreviations used are: aqueous (aq.), hours (h), minutes (min), microwave (MW), room temperature (rt), on (overnight), saturated (sat.).

5.0 Synthesis and characterization of compounds 9–23.

5.1 Synthetic schemes

Scheme S1. Synthesis of **9**–**17**.

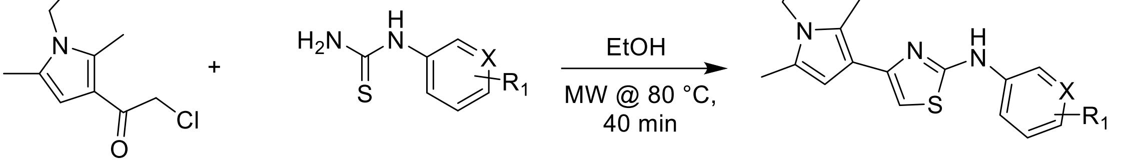

9a, X = CH, R_1 = 3-CF_3
10a, X = CH, R_1 = 2-CF_3
11a, X = CH, R_1 = 4-CF_3
12a, X = CH, R_1 = H
13a, X = CH, R_1 = 4-$N(CH_3)_2$
14a, X = CH, R_1 = 4-OCH_3
15a, X = CH, R_1 = 4-Cl
16a, X = N, R_1 = 4-Cl
17a, X = CH, R_1 = 3,5-Cl

9, X = CH, R_1 = 3-CF_3 (Y = 20%)
10, X = CH, R_1 = 2-CF_3 (Y = 31%)
11, X = CH, R_1 = 4-CF_3 (Y = 40%)
12, X = CH, R_1 = H (Y = 41%)
13, X = CH, R_1 = 4-$N(CH_3)_2$ (Y = 40%)
14, X = CH, R_1 = 4-OCH (Y = 44%)
15, X = CH, R_1 = 4-Cl (Y = 73%)
16, X = N, R_1 = 4-Cl (Y = 76%)
17, X = CH, R_1 = 3,5-Cl (Y = 41%)

Scheme S2. Synthesis of **18**.

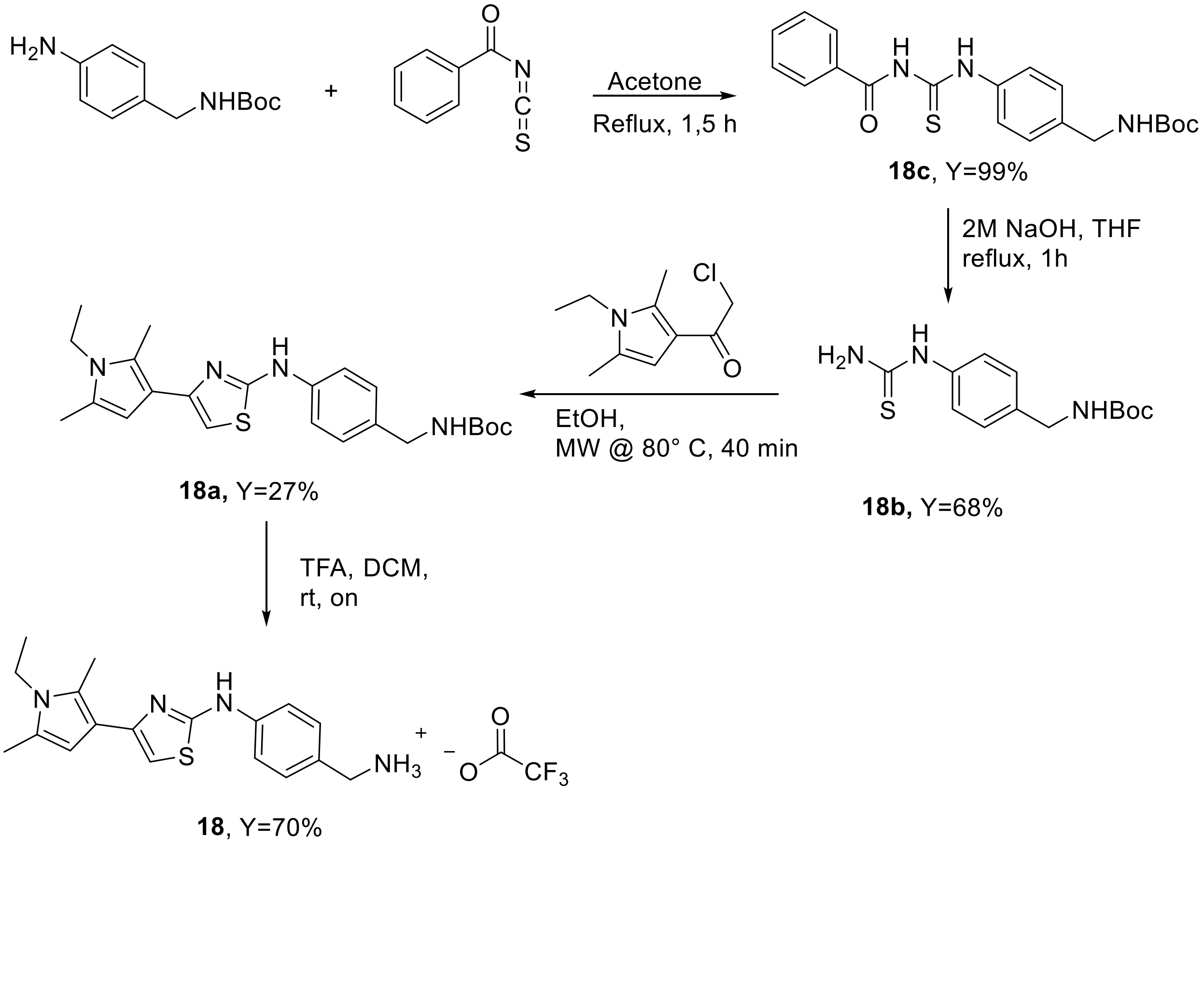

Scheme S3. Synthesis of **19** and **20**.

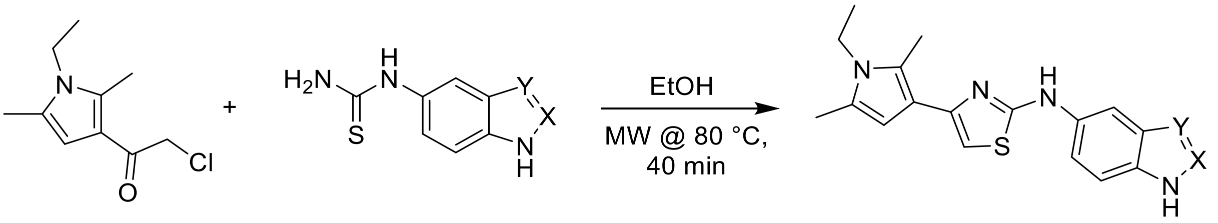

19a, X = CH, Y = N
20a, X = N, Y = CH

19, X = CH, Y = N (Y=10%)
20, X = N, Y = CH (Y=7%)

Scheme S4. Synthesis of **21**.

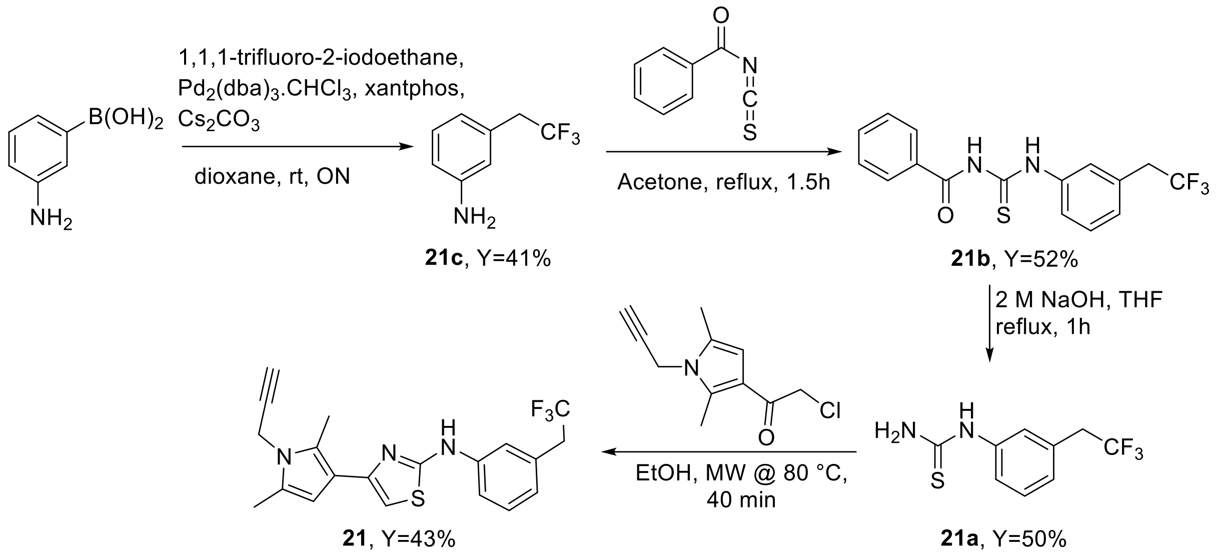

Scheme S5. Synthesis of **22** and **23**.

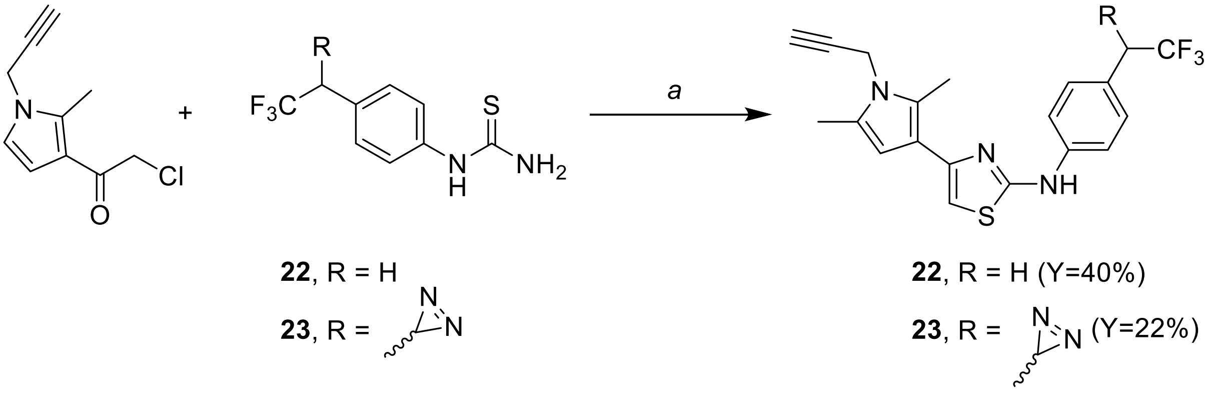

Reagent and conditions: *a*) EtOH, MW @ 80 °C, 40 min (Y=40%) for compound **22**; EtOH, 50 °C for 3 h (Y=22%) for compound **23**.

5.2 General procedures

General procedure 1 (GP1): **thiazoles synthesis**

Phenyl thiourea (1 eq.) and 3-chloroacetyl pyrroles (1 eq.) were dissolved in EtOH (0.2 M) and heated under microwave irradiation for 40 min at 80 °C. The reaction mixture was cooled and the solvent removed under reduced pressure. The crude product was washed several times with isopropanol and the precipitate yielded the pure compound.

General procedure 2 (GP2): ***N*-(Phenylcarbamothioyl)-benzamide synthesis**

To a stirred solution of aniline (1 eq.) in acetone (0.2 M), benzoyl isothiocyanate (1 eq.) was added and the reaction mixture was stirred to reflux for 1.5 h. The mixture was then poured into ice to obtain the desired product as precipitate.

General procedure 3 (GP3): **Phenylthiourea synthesis**

A solution of phenyl carbamothioyl benzamide (1 eq.) and 2M NaOH solution in THF (0.1 M) is refluxed for 1 hour. The reaction mixture was cooled to RT, acidified with aq. HCl 0.1 M and filtered off to release the desired product.

5.3 Synthesis and Characterization of compounds 9–23.

4-(1-Ethyl-2,5-dimethyl-1*H*-pyrrol-3-yl)-*N*-(3-(trifluoromethyl)phenyl)thiazol-2-amine (9)

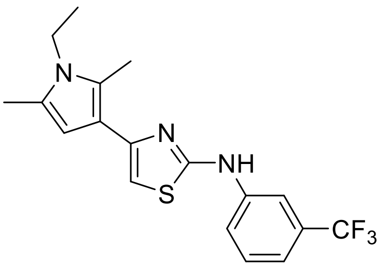

According to GP1, 2-chloro-1-(1-ethyl-2,5-dimethyl-1*H*-pyrrol-3-yl)ethan-1-one (0.200 g, 1 mmol) and 1-(3-(trifluoromethyl)phenyl)thiourea (0.215 g, 1 mmol) in EtOH (5 mL) were used to obtain **9** as dark green powder (0.073 g, 20%). **^{1}H NMR (500 MHz, DMSO–*d*$_6$)** δ 10.56 (s, 1H), 8.54 (s, 1H), 7.63 (d, *J* = 8.6, 1H), 7.51 (t, *J* = 7.9, 1H), 7.25 (d, *J* = 7.6, 1H), 6.59 (s, 1H), 6.04 (s, 1H), 3.83 (q, *J* = 7.1, 2H), 2.53 (s, *J* = 6.8,

4H), 2.18 (s, 3H), 1.17 (t, J = 7.2, 3H). **^{13}C NMR (126 MHz DMSO–d_6)** 161.72, 148.28, 141.98, 129.89, 129.74 (q, J_{CF} = 31.0, C_{quat}), 126.29, 125.45, 124.32, 120.25, 116.91 (d, J = 3.7), 114.20, 112.52 (d, J = 4.0), 105.01, 98.48, 37.50, 15.93, 11.89, 10.90. **^{19}F NMR (470 MHz, DMSO)** δ -61.28 (s, CF_3). **HR–MS (ESI$^+$)** *m/z* calcd for $C_{18}H_{19}F_3N_3S$ [*M*+H]$^+$: 366.12463, found: 366.12351.

4-(1-Ethyl-2,5-dimethyl-1*H*-pyrrol-3-yl)-*N*-(2-(trifluoromethyl)phenyl)thiazol-2-amine (10)

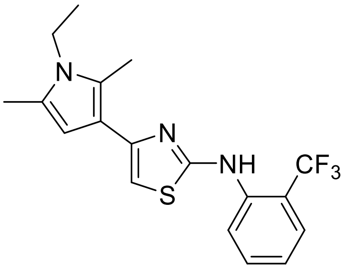

According to GP1, 2-chloro-1-(1-ethyl-2,5-dimethyl-1*H*-pyrrol-3-yl)ethan-1-one (0.100 g, 0.501 mmol) and 1-(2-(trifluoromethyl)phenyl)thiourea (0.110 g, 0.501 mmol) in EtOH (2.5 mL) were used to obtain **10** as light beige powder, (0.114 g, 31%). **^{1}H NMR (500 MHz, DMSO–d_6)** δ 9.52 (br s, 1H), 7.98-8.13 (m, 1H), 7.73 (d, J = 7.8, 1H), 7.67 (br t, J = 7.5, 1H), 7.33 (br t, J = 7.5, 1H), 6.51 (br s, 1H), 5.96-5.99 (m, 1H), 3.80 (q, J = 7.2, 2H), 2.37 (s, 3H), 2.17 (s, 3H), 1.14 ppm (t, J = 7.2, 3H). **^{13}C NMR (126 MHz DMSO–d_6)** 166.2, 138.0, 133.9, 126.9, 126.5, 125.9, 125.3, 124.8, 122.6, 111.5, 105.2, 99.2, 37.7, 15.9, 11.8, 10.9 ppm. **^{19}F NMR (470 MHz, DMSO–d_6)** δ -59.41 (s, 3F). **HR-MS (ESI$^+$)** *m/z* calcd for $C_{18}H_{19}F_3N_3S$ [*M*+H]$^+$: 366.12463, found: 366.12386.

4-(1-Ethyl-2,5-dimethyl-1*H*-pyrrol-3-yl)-*N*-(4-(trifluoromethyl)phenyl)thiazol-2-amine (11)

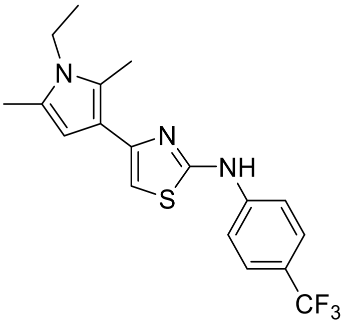

According to GP1, 2-chloro-1-(1-ethyl-2,5-dimethyl-1*H*-pyrrol-3-yl)ethan-1-one (0.150 g, 0.75 mmol) and 1-(4-(trifluoromethyl)phenyl)thiourea (0.166 g, 0.75 mmol) in EtOH (3.7 mL) were used to give **11** as yellowish powder (0.110 g, 40%). **^{1}H NMR (500 MHz, DMSO–*d*$_6$)** 10.58 (s, 1H), 7.88 (d, *J* = 8.6, 2H), 7.65 (d, *J* = 8.7, 2H), 6.60 (s, 1H), 6.05 (s, 1H), 3.83 (q, *J* = 7.2, 2H), 2.49 (s, 3H), 2.19 (s, 3H), 1.18 (t, *J* = 7.2 Hz, 3H). **^{13}C NMR (126 MHz DMSO–*d*$_6$)** 161.34, 148.37, 144.67, 126.24 (q, *J* = 3.6 2 x Ar*C*H), 126.13, 125.8, 123.64, 120.50 (q, J_{CF} = 31.9, C_{quat}), 116.26 (2 x Ar*C*H), 114.32, 105.25, 98.98, 37.52, 15.93, 11.88, 11.08. **^{19}F NMR (470 MHz, DMSO–*d*$_6$)** δ -59.77 (s, C*F*$_3$). **HR–MS (ESI$^+$)** *m/z* calcd for $C_{18}H_{18}F_3N_3S$ [*M*+H]$^+$: 366.12463, found: 366.12389.

4-(1-Ethyl-2,5-dimethyl-1*H*-pyrrol-3-yl)-*N*-phenylthiazol-2-amine (12)

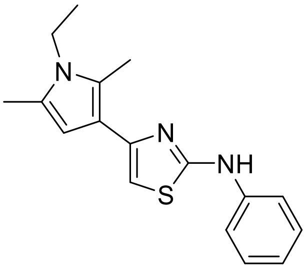

According to GP1, 2-chloro-1-(1-ethyl-2,5-dimethyl-1*H*-pyrrol-3-yl)ethan-1-one (0.150 g, 0.75 mmol) and 1-phenylthiourea (0.114 g, 0.75 mmol) in EtOH (3.7 mL) were used to give **12** as beige powder (0.90 g, 41%).**^{1}H NMR (500 MHz, DMSO–*d*$_6$)** δ 10.45 (s, 1H), 7.63 (d, *J* = 8.0, 2H), 7.34 (t, *J* = 7.8, 2H), 7.01 (t, *J* = 7.2, 1H), 6.52 (s, 1H), 6.04 (s, 1H), 3.84 (q, *J* = 7.1, 2H), 2.47 (s, 3H), 2.19 (s, 3H), 1.17 (t, *J* = 7.2, 3H). **^{13}C NMR (126 MHz DMSO–*d*$_6$)** 163.9, 140.7, 129.1, 126.310, 124.715, 122.1, 117.710, 113.1, 105.1, 97.8, 37.6, 15.9, 11.8, 11.0. **HR–MS (ESI$^+$)** *m/z* calcd for $C_{17}H_{20}N_3S$ [*M*–H]$^+$: 298.13724, found: 298.13645.

N^1-(4-(1-Ethyl-2,5-dimethyl-1*H*-pyrrol-3-yl)thiazol-2-yl)-N^4,N^4-dimethylbenzene-1,4-diamine (13)

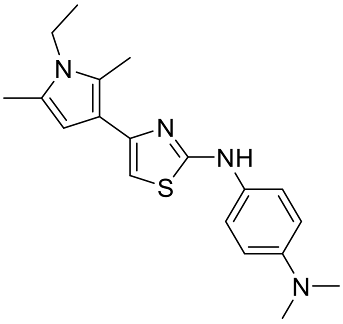

According to GP1, 2-chloro-1-(1-ethyl-2,5-dimethyl-1*H*-pyrrol-3-yl)ethan-1-one (0.200 g, 1 mmol) and 1-(4-(dimethylamino)phenyl)thiourea (0.195 g, 1 mmol) in EtOH (5 mL) were used to give **13** as grey powder (0.14 g, 40%). **^{1}H NMR (500 MHz, DMSO‒d_6)** δ 10.48 (s, 1H), 7.79 (m, 2H), 7.62 (m, 2H), 6.55 (s, 1H), 6.05 (s, 1H), 3.84 (dd, *J* = 13.5, 6.4, 2H), 3.08 (s, 6H), 2.53 (s, 3H), 2.19 (s, 3H), 1.18 (*t*, J = 7.0, 3H). **^{13}C NMR (126 MHz DMSO‒d_6)** δ 163.5, 147.6, 136.2, 126.2, 124.4, 120.6, 117.5, 114.1, 105.2, 98.3, 45.1, 37.5, 15.9, 11.8, 11.1. **HR‒MS (ESI$^+$)** *m/z* calcd for $C_{19}H_{25}N_4S$ [*M*+H]$^+$: 341.17944, found: 341,17848.

4-(1-Ethyl-2,5-dimethyl-1*H*-pyrrol-3-yl)-*N*-(4-methoxyphenyl)thiazol-2-amine (14)

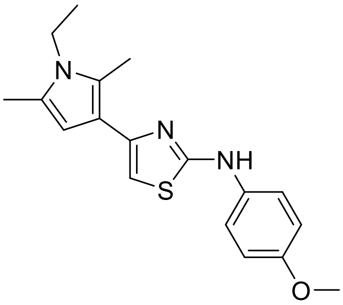

According to GP1, 2-chloro-1-(1-ethyl-2,5-dimethyl-1*H*-pyrrol-3-yl)ethan-1-one (0.200 g, 1 mmol) and 1-(4-methoxyphenyl)thiourea (0.182 g, 1 mmol) in EtOH (5 mL) were used to give **14** as brown powder (0.144 g, 44%). **^{1}H NMR (500 MHz, DMSO‒d_6)** δ 10.47 (s, 1H), 7.51 (d, *J* = 8.9, 2H), 6.97 (d, *J* = 8.9, 2H), 6.48 (s, 1H), 6.03 (s, 1H), 3.84 (q, *J* = 14.3, 7.1, 2H), 3.75 (s, 3H), 2.42 (s, 3H), 2.19 (s, 3H), 1.17 (t, *J* = 7.2, 3H). **^{13}C NMR (126 MHz DMSO‒d_6)** δ 167.2, 157.1, 138.9, 131.5, 126.9, 126.0, 122.8, 114.9, 109.3, 104.9, 97.1, 55.4, 37.8, 15.8, 11.8, 10.9. **HR‒MS (ESI$^+$)** *m/z* calcd for $C_{18}H_{22}N_3OS$ [*M*+H]$^+$: 328.14781, found: 328.14684.

N-(4-Chlorophenyl)-4-(1-ethyl-2,5-dimethyl-1H-pyrrol-3-yl)thiazol-2-amine (15)

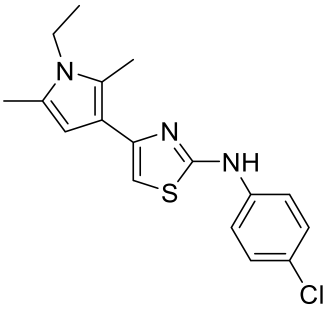

According to GP1, 2-chloro-1-(1-ethyl-2,5-dimethyl-1*H*-pyrrol-3-yl)ethan-1-one (0.150 g, 0.75 mmol) and 1-(4-chlorophenyl)thiourea (0.14 g, 0.75 mmol) in EtOH (3,7 mL) were used to give **15** as yellowish powder (0.18 g, 73%). **^{1}H NMR (500 MHz, DMSO–*d*$_6$)** *δ* 10.51 (s, 1H), 7.73 – 7.63 (m, 2H), 7.41 – 7.28 (m, 2H), 6.53 (s, 1H), 6.03 (s, 1H), 3.83 (q, *J* = 7.1, 2H), 2.46 (s, 3H), 2.18 (s, 3H), 1.17 (t, *J* = 7.2, 3H). **^{13}C NMR (126 MHz DMSO–*d*$_6$)** 162.4, 146.9, 139.9, 128.8, 126.2, 124.8, 124.5, 118.7, 113.6, 105.2, 98.2, 37.5, 15.9, 11.8, 11.0. **HR–MS (ESI$^+$)** *m/z* calcd for $C_{17}H_{19}ClN_3S$ [*M*+H]$^+$: 332,09827, found: 332.09257.

N-(6-Chloropyridin-3-yl)-4-(1-ethyl-2,5-dimethyl-1*H*-pyrrol-3-yl)thiazol-2-amine (16)

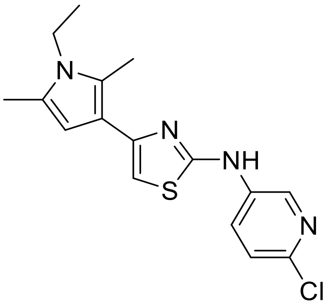

According to GP1, 2-chloro-1-(1-ethyl-2,5-dimethyl-1*H*-pyrrol-3-yl)ethan-1-one (0.150 g, 0.75 mmol) and 1-(6-chloropyridin-3-yl)thiourea (0.141 g, 0.75 mmol) in EtOH (3.7 mL) were used to give **16** as yellowish powder (0.190 g, 76%). **^{1}H NMR (500 MHz, DMSO–*d*$_6$)** *δ* 10.47 (s, 1H), 8.77 (d, *J* = 2.7, 1H), 8.17 (dd, *J* = 8.7, 2.9, 1H), 7.45 (d, *J* = 8.7, 1H), 6.59 (s, 1H), 6.04 (d, *J* = 0.8, 1H), 3.83 (q, *J* = 7.2, 2H), 2.48 (s, 3H), 2.19 (s, 3H), 1.17 (t, *J* = 7.2, 3H). **^{13}C NMR (126 MHz DMSO–*d*$_6$)** 161.4, 148.2, 140.8, 137.9, 137.6, 126.9, 126.2, 124.3, 124.2, 114.2, 105.2, 99.0, 37.5, 16.0, 11.9, 11.1. **HR–MS (ESI$^+$)** *m/z* calcd for $C_{16}H_{18}ClN_4S$ [*M*+H]$^+$: 333.09352, found: 333.09257.

N-(3,5-diChlorophenyl)-4-(1-ethyl-2,5-dimethyl-1H-pyrrol-3-yl)thiazol-2-amine (17)

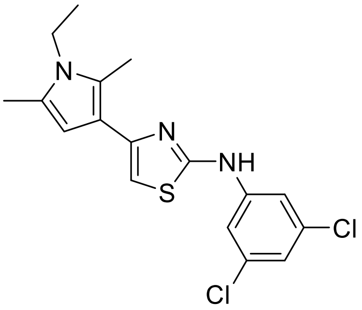

According to GP1, 2-chloro-1-(1-ethyl-2,5-dimethyl-1*H*-pyrrol-3-yl)ethan-1-one (0.200 g, 1 mmol) and 1-(3,5-dichlorophenyl)thiourea (0.22 g, 1 mmol) in EtOH (5 mL) were used to give **17** as greenish powder (0.07 g, 41%). **^{1}H NMR (500 MHz, DMSO–*d*$_6$)** *δ* 10.63 (s, 1H), 7.83 (d, *J* = 1.8, 2H), 7.08 (t, *J* = 1.8, 1H), 6.62 (s, 1H), 6.03 (s, 1H), 3.83 (q, *J* = 7.2, 2H), 2.54 (s, 3H), 2.18 (s, 3H), 1.17 (t, *J* = 7.2, 3H). **^{13}C NMR (126 MHz, DMSO–*d*$_6$)** *δ* 161.2, 148.4, 143.4, 134.2, 126.3, 124.3, 119.5, 114.7, 114.2, 104.9, 99.0, 37.5, 15.9, 11.9, 11.04. **HR-MS (ESI$^+$)** *m/z* calcd for $C_{17}H_{18}Cl_2N_3S$ [*M*+H]$^+$: 366.05930, found: 366.05843.

(4-((4-(1-Ethyl-2,5-dimethyl-1H-pyrrol-3-yl)thiazol-2-yl)amino)phenyl)methanaminium formate (18)

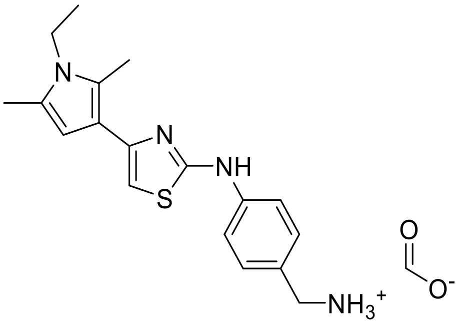

To a stirred solution of **18a** (0.05 g, 0.12 mmol) in DCM (5 mL), TFA (15 eq.) was slowly added at 0 ° C. The mixture was stirred at rt on. Once the reaction was completed, the solvent was concentrated and the crude mixture was purified with prep. HPLC to afford **18** as dark green powder (0.03 g, 70%). **^{1}H NMR (500 MHz, DMSO–*d*$_6$)** *δ* 10.20 (s, 1H), 8.30 (s, 2H), 7.69 (d, *J* = 8.5, 2H), 7.36 (d, *J* = 8.5, 2H), 6.52 (s, 1H), 6.03 (s, 1H), 3.90 (s, 3H), 3.83 (q, *J* = 7.2, 2H), 2.54 (s, 3H), 2.19 (s, 3H), 1.17 (t, *J* = 7.2 Hz, 3H). **^{13}C NMR (126 MHz, DMSO–*d*$_6$)** *δ* 164.8, 162.3, 162.0, 148.8, 141.8, 129.9, 126.5, 124.6, 116.8, 114.9, 105.6, 98.4, 49.0, 42.9, 16.4, 12.3, 11.4. **HR-MS (ESI$^+$)** *m/z* calcd for $C_{18}H_{23}N_4S$ [*M*–H]$^+$: 327.16434, found: 327.16321.

_N_-(1_H_-Benzo[_d_]imidazol-5-yl)-4-(1-ethyl-2,5-dimethyl-1_H_-pyrrol-3-yl)thiazol-2-amine (19)

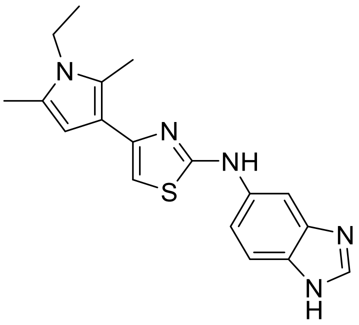

According to GP1, 2-chloro-1-(1-ethyl-2,5-dimethyl-1*H*-pyrrol-3-yl)ethan-1-one (0.150 g, 0.75 mmol) and 1-(1*H*-benzo[*d*]imidazol-5-yl)thiourea (0.150 g, 0.75 mmol) in EtOH (5 mL) were used to give **19** as white powder (0.020 g, 10%). **^{1}H NMR (500 MHz, DMSO–d_6)** δ 14.62 (s, 1H), 10.52 (s, 1H), 9.36 (s, 1H), 8.64 (d, *J* = 1.6 Hz, 1H), 7.75 (d, *J* = 8.9 Hz, 1H), 7.48 (dd, *J* = 8.9, 1.9 1H), 6.57 (s, 1H), 6.09 (s, 1H), 3.85 (q, *J* = 7.1, 2H), 2.53 (s, 3H), 2.21 (s, 3H), 1.20 (t, *J* = 7.2, 3H). **^{13}C NMR (500 MHz DMSO–d_6)** 161.7, 148.4, 139.8, 139.5, 131.8, 126.1, 125.4, 124.4, 116.8, 114.9, 114.5, 105.4, 99.8, 98.4, 25.5, 16.0, 11.9, 11.3. **HR-MS (ESI$^+$)** *m/z* calcd for $C_{18}H_{20}N_5S$ [*M*–H]$^+$: 338.14394, found: 338.14203.

4-(1-Ethyl-2,5-dimethyl-1_H_-pyrrol-3-yl)-_N_-(1_H_-indazol-5-yl)thiazol-2-amine (20)

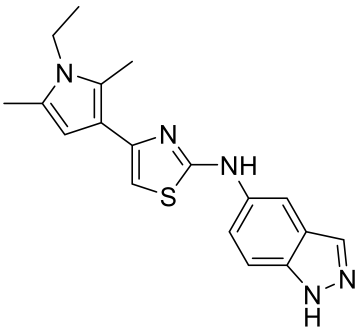

According to GP1, 2-chloro-1-(1-ethyl-2,5-dimethyl-1*H*-pyrrol-3-yl)ethan-1-one (0.150 g, 0.75 mmol) and 1-(1*H*-indazol-5-yl)thiourea (0.150 g, 0.75 mmol) in EtOH (4 mL) were used to give **20** as beige powder (0.015 g, 7%). **^{1}H NMR (500 MHz, DMSO–d_6)** δ 12.84 (s, 1H), 10.22 (s, 1H), 8.33 (s, 1H), 7.91 (s, 1H), 7.62 (d, *J* = 8.7, 1H), 7.04 (dd, *J* = 8.7, 1.7, 1H), 6.52 (s, 1H), 6.07 (s, 1H), 3.85 (q, *J* = 7.2, 2H), 2.55 (s, 3H), 2.21 (s, 3H), 1.20 (t, *J* = 7.2, 3H). **^{13}C NMR (126 MHz, DMSO–d_6)** δ 161.9, 148.5, 140.9, 139.7, 133.3, 125.9, 124.4, 120.6, 117.7, 114.6, 113.1, 105.3, 97.9, 95.3, 37.5, 15.9, 11.9, 11.2. **HR-MS (ESI$^+$)** *m/z* calcd for $C_{18}H_{20}N_5S$ [*M*–H]$^+$: 338.14394, found: 338.14212.

4-(2,5-Dimethyl-1-(prop-2-yn-1-yl)-1*H*-pyrrol-3-yl)-*N*-(3-(2,2,2-trifluoroethyl)phenyl)thiazol-2-amine (21)

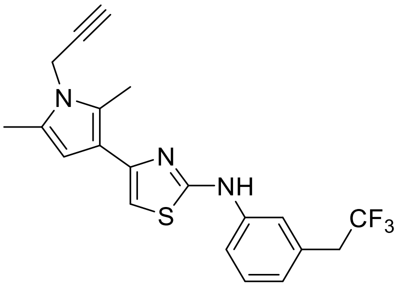

According to GP1, 2-chloro-1-(2,5-dimethyl-1-(prop-2-yn-1-yl)-1*H*-pyrrol-3-yl)ethan-1-one (CAS = 1155980-26-9) (0.036 g, 0.17 mmol) and **21a** (0.04 g, 0.17 mmol) in EtOH (0,85 mL) were used to give **21** as beige powder (0.03 g, 43%). **^{1}H NMR (500 MHz, DMSO–*d₆*)** δ 10.22 (s, 1H), 7.83 (s, 1H), 7.57 (d, *J* = 7.5, 1H), 7.31 (t, *J* = 7.6, 1H), 6.93 (d, *J* = 6.8, 1H), 6.58 (s, 1H), 6.08 (s, 1H), 4.68 (s, 2H), 3.61 (m, Ar*C*H_2CF_3,2H), 3.33 (s, 1H), 2.56 (2, 3H), 2.22 (s, 3H). **^{13}C NMR (126 MHz, DMSO–*d$_6$*)** δ 162.8, 141.9, 131.6, 129.6, 127.9, 127.3, 125.6, 125.4, 123.5, 118.9, 116.8, 115.0, 105.9, 99.0, 80.1, 74.8, 32.82, 12.35, 11.49. **^{19}F NMR (470 MHz, DMSO–*d$_6$*)** δ -64.21 (t, *J* = 11.4). **HR-MS (ESI$^+$)** *m/z* calcd for $C_{20}H_{19}F_3N_3S$ [*M*–H]$^+$: 390.12518, found: 390.12415.

4-(2,5-Dimethyl-1-(prop-2-yn-1-yl)-1*H*-pyrrol-3-yl)-*N*-(4-(2,2,2-trifluoroethyl)phenyl)thiazol-2-amine (22)

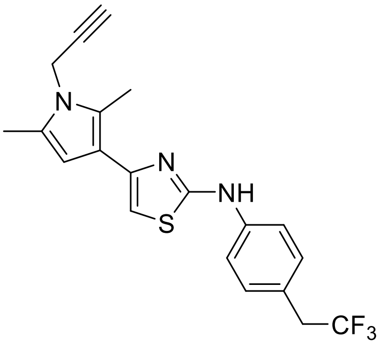

According to GP1, 2-chloro-1-(2,5-dimethyl-1-(prop-2-yn-1-yl)-1*H*-pyrrol-3-yl)ethan-1-one (CAS = 1155980-26-9) (0.100 g, 0.43 mmol) and 1-(4-(2,2,2-trifluoroethyl)phenyl)thiourea (0.09 g, 0.43 mmol) in EtOH (2.15 mL) were used to give **22** as brownish (0,07g, 40%). **^{1}H NMR (500 MHz, DMSO–*d$_6$*)** δ 10.30 (s, 1H), 7.67 (d, *J* = 8.6, 2H), 7.28 (d, *J* = 8.4, 2H), 6.57 (s, 1H), 6.07 (s, 1H), 4.68 (d, *J* = 2.4, 2H), 3.54 (m, 2H, overlapping with water DMSO), 3.33 (t, *J* = 2.4, 1H), 2.54 (s, 3H), 2.22 (s, 3H). **^{13}C NMR (500 MHz DMSO–*d$_6$*)** δ 162.4, 140.9, 130.9, 126.9, 125.0, 116.9, 114.6,

105.6, 98.7, 79.7, 74.5, 37.9 (q, *J* = 28.4, Ar*C*H_2CF_3), 32.4, 11.9, 11.2. **^{19}F NMR (470 MHz, DMSO–*d*$_6$)** *δ* -64.71 (t, *J* = 11.5). **HR-MS (ESI$^+$)** *m/z* calcd for $C_{20}H_{19}F_3N_3S$ [*M*–H]$^+$: 390.12518, found: 390.12387.

4-(2,5-Dimethyl-1-(prop-2-yn-1-yl)-1*H*-pyrrol-3-yl)-*N*-(4-(3-(trifluoromethyl)-3*H*-diazirin-3-yl)phenyl)thiazol-2-amine (23)

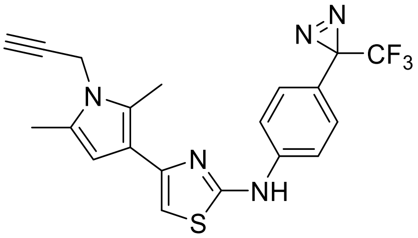

In a dark dry flask, 2-chloro-1-(2,5-dimethyl-1-(prop-2-yn-1-yl)-1*H*-pyrrol-3-yl)ethan-1-one (CAS = 1155980-26-9) (0.035 g, 0.167 mmol) and 1-(4-(3-(trifluoromethyl)-3*H*-diazirin-3-yl)phenyl)thiourea (CAS = 2377034-45-0) (0.044 g, 0.044 mmol) in EtOH (1.5 mL) were heated to 50 °C for 3 h. After the reaction was completed, EtOH was evaporated, and the crude product was washed several times with isopropanol to release **23** as greenish powder (0,015 g, 22%). **^{1}H NMR (500 MHz, DMSO–*d*$_6$)** *δ* 10.44 (s, 1H), 7.81 (d, *J* = 8.8, 2H), 7.23 (d, *J* = 8.5, 2H), 6.62 (s, 1H), 6.08 (s, 1H), 4.68 (d, *J* = 2.1, 2H), 3.33 (t, *J* = 2.1, 1H), 2.52 (s, 3H), 2.22 (s, 3H). **^{13}C NMR (126 MHz DMSO–*d*$_6$)** *δ* 161.7, 147.4, 142.9, 127.4, 126.8, 125.0, 121.0, 119.2, 117.0, 114.6, 105.6, 99.4, 79.6, 74.4, 32.4, 11.8, 11.2. **^{19}F NMR (470 MHz, DMSO–*d*$_6$)** *δ* -64,75 (s, C*F*$_3$). **HR-MS (ESI$^+$)** *m/z* calcd for $C_{20}H_{17}F_3N_5S$ [*M*+H]$^+$: 416.11568, found: 416.11416.

***Tert*-butyl (4-((4-(1-ethyl-2,5-dimethyl-1*H*-pyrrol-3-yl)thiazol-2-yl)amino)benzyl)carbamate (18a)**

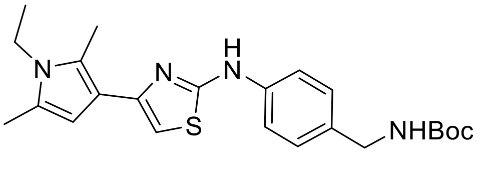

According to GP1, 2-chloro-1-(2,5-dimethyl-1-(prop-2-yn-1-yl)-1*H*-pyrrol-3-yl)ethan-1-one (0.106 g, 0.53 mmol) and **18b** (0.15 g, 0.53 mmol) in EtOH (2.65 mL) were used to give **18a** as dark green powder (0,06 g, 27%). **^{1}H NMR (500 MHz, DMSO–*d*$_6$)** *δ* 11.00 (s, 1H), 8.75 (s, 1H), 7.98 (d, *J* = 6.9, 2H), 7.65 (d, *J* = 7.0, 2H), 6.93 (s, 1H),

6.46 (s, 1H), 4.51 (s, 2H), 4.26 (m, 2H), 2.87 (s, 3H), 2.61 (s, 3H), 1.81 (s, 9H), 1.59 (m, 3H). **^{13}C NMR (500 MHz DMSO–d_6)** δ 163.6, 156.0, 141.5, 139.5, 130.1, 128.4, 128.0, 126.5, 124,9, 117.9, 105.3, 97.8, 78.0, 43.1, 37.7, 28.4, 16.0, 11.9, 11.1.

***Tert*-butyl (4-thioureidobenzyl)carbamate (18b)**

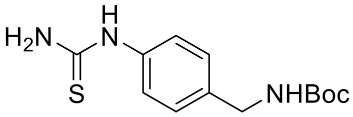

According to GP3, using **18c** (0.30 g, 0,84 mmol) 2M NaOH (2 mL) in THF (8.4 mL) to give, after acidification and filtration **18b** as white solid (0,16 g, 68%). **^{1}H NMR (500 MHz, DMSO–d_6)** δ 9.63 (s, 1H), 7.37 (t, J = 6.1, 1H), 7.31 (d, J = 8.2, 2H), 7.17 (d, J = 8.3, 2H), 4.08 (d, J = 6.2, 2H), 1.39 (s, 9H). **^{13}C NMR (500 MHz DMSO–d_6)** δ 180.9, 155.8, 137.6, 136.4, 127.2, 123.1, 77.7, 42.9, 28.3.

***Tert*-butyl (4-(3-phenylthioureido)benzyl)carbamate (18c)**

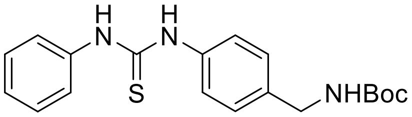

According to GP2, *tert*-butyl (4-aminobenzyl)carbamate (0.60 g, 2.7 mmol) and benzoyl isothiocyanate (0.36 mL, 2.7 mmol) in acetone (13.5 mL) yielded **18c** as yellowish solid (0.96 g, 99%). **^{1}H NMR (500 MHz, DMSO–d_6)** δ 12.57 (s, 1H), 11.59 (s, 1H), 7.97 (d, J = 7.4, 2H), 7.66 (t, J = 7.4, 1H), 7.61 (d, J = 8.3, 2H), 7.54 (t, J = 7.8, 2H), 7.46 (t, J = 6.2, 1H), 7.27 (d, J = 8.3, 2H), 4.13 (d, J = 6.1, 2H), 1.40 (s, 9H). **^{13}C NMR (500 MHz DMSO–d_6)** δ 179.1, 168.3, 155.8, 138.4, 136.5, 133.2, 132.2, 128.7, 128.5, 127.2, 124.3, 77.8, 42.9, 28.3.

1-(3-(2,2,2-Trifluoroethyl)phenyl)thiourea (21a)

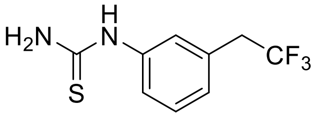

According to GP3, using **21b** (0.15 g, 0,44 mmol) and 2M NaOH (1.5 mL) in THF (4,4 mL) to give after acidification and filtration **21a** as beige powder (0.05 g, 50%). **^{1}H NMR (500 MHz, DMSO–d_6)** δ 9.75 (s, 1H), 7.51–7.27 (m, 2H), 7.10 (d, J = 5.1, 1H), 3.62 (d, J = 11.0, 2H). **^{13}C NMR (126 MHz, DMSO–d_6)** δ 181.1, 139.4, 130.8 (dd, J = 5.0, 2.2) 128.8, 126.2, 124.6, 122.6, 38.3 (q, J = 28.4, CH_2CF_3). **^{19}F NMR (470 MHz, $CDCl_3$)** δ -64.21.

***N*-((3-(2,2,2-Trifluoroethyl)phenyl)carbamothioyl)benzamide (21b)**

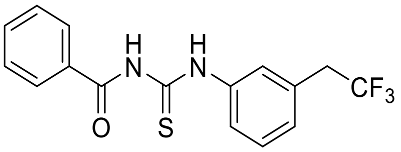

According to GP 2, using **21c** (0.200 g, 1.03 mmol) and benzoyl isothiocyanate (0.13 mL) in acetone (5.5 mL) to give **21b** as yellow powder (0.18 g, 52%). **^{1}H NMR (500 MHz, DMSO–*d*$_6$)** δ 12.63 (s, 1H), 11.60 (s, 1H), 7.98 (m, 2H), 7.79 – 7.70 (m, 1H), 7.66 (m, 2H), 7.54 (m, 2H), 7.44 (t, *J* = 7.7, 1H), 7.27 (d, *J* = 7.0, 1H), 3.70 (dd, *J* = 22.8, 11.3, ArC*H*$_2$CF$_3$, 2H). **^{13}C NMR (126 MHz, DMSO–*d*$_6$)** δ 179.2, 168.3, 138.2, 133.2, 132.15, 131.0 (dd, *J* = 5.3, 2.7, Ar*C*), 129.3, 128.8, 128.7, 128.5, 128.2, 126.0, 123.9, 38.2 (q, *J* = 28.5, *C*H$_2$CF$_3$). **^{19}F NMR (470 MHz, CDCl$_3$)** δ -65.73.

3-(2,2,2-Trifluoroethyl)aniline (21c)

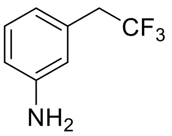

To a solution of (3-aminophenyl)boronic acid (0.50 g, 3.65 mmol) and 1,1,1-trifluoro-2-iodoethane (1.53 g, 7.3 mmol) dissolved in dioxane (9 mL), a mixture of $Pd_2(dba)_3 \cdot CHCl_3$ (0.189 g, 0.18 mmol), xantphos (0.36 g, 0.62 mmol) and Cs_2CO_3 (4.75 g, 14.6 mmol) in dioxane (18 mL) were added. After 1 minute, H_20 (1.25 mL) was added and the reaction mixture was stirred at rt. The suspension was washed with H_2O and extracted with EtOAc (3 x 20 mL). The combined organic layers were dried over Na_2SO_4, filtered, concentrated in vacuo and purified by flash column chromatography (40% EtOAc in Hexane) to afford **21c** as yellow oil (0.26 g, 41%). **^{1}H NMR (500 MHz, CDCl$_3$)** δ 7.13 (t, *J* = 7.8, 1H), 6.69–6.64 (m, 2H), 6.61 (s, 1H), 3.62 (br, 2H), 3.27 (q, *J* = 10.9, 2H). **^{13}C NMR (126 MHz, CDCl$_3$)** δ 146.7, 131.3 (dd, *J* = 5.3, 2.5, Ar*C*), 129.7, 125.9 (*C*F$_3$), 120.5, 116.8, 114.9, 40.3 (q, *J* = 29.6, *C*H$_2$CF$_3$). **^{19}F NMR (470 MHz, CDCl$_3$)** δ -65.71 (s, *C*F$_3$).

6.0 *In vitro* drug sensitivity assay on *Plasmodium falciparum* asexual blood stage parasites

Plasmodium falciparum NF54 parasites in the asexual blood stage were cultured in RPMI 1640 medium. This was supplemented with 25 mM sodium bicarbonate (pH 7.3), 100 µg/mL neomycin, 0.5% Albumax II, and 0.36 mM hypoxanthine. In order to test

the compound activity against *P. falciparum* NF54 asynchronous parasites, the [3*H*]-hypoxanthine incorporation assay was used as described.[4] Compounds dissolved in DMSO (10 mg/mL) are diluted in hypoxanthine - free culture media and was titrated in duplicates over a 64-fold range in 96 well plates. Parasite culture diluted to 0.3% parasitemia with 1.25% final haematocrit with hypoxantine-free medium was added at 100 µL to each well. Following a 48 h incubation, 0.25 µM [3*H*]-hypoxanthine was added per well. Plates are incubated for another 24 h. Radioactivity was then measured using MicroBeta2 liquid scintillation counter (Perkin Elmer, Waltham, US) following parasite harvesting onto Microbeta FilterMate cell harvester (Perkin, Elmer, Waltham, US). The fifty percent inhibitory concentration (IC_{50}) was then calculated by linear interpolation as described.[5]

7.0 Analytical SDS-PAGE

7.1 Background lysate

For the background lysate, HEK293 cells (passage 27) were grown to 90% confluency in Dulbecco´s Modified Eagle´s Medium (DMEM high glucose, Sigma) supplemented with 2 mM L-glutamine and 10% (v/v) heat-inactivated fetal calf serum (FCS) in T-175 cell culture flasks. Lysis was performed using HEPES buffer (pH 7.5, Roth) containing 1% Nonidet P 40 alternative (NP-40), 0.4% sodium dodecyl sulfate (SDS, Roth) and 1% sodium deoxycholate (Roth). The lysate was gently sonicated three times for 10 s on a Bandelin Sonoplus HD2070 at 10% amplitude. The lysate was cleared by centrifugation at 16.000 x g for 10 min. The protein content of the supernatant was determined using a BCA assay (ROTIQuant, Roth) according to the suppliers specifications and adjusted to 5 µg/µL to yield the stock solutions used in the experiments.

7.2 Reaction with probe 23

The protein samples were prepared to result in 50 µg lysate protein per lane and/or 10 µg of the respective recombinant protein (see section 1 of this SI). For heat denaturation controls, samples were heated to 95 °C for 5 min and cooled down on ice before the reaction with the probe. In case of competition experiments, compound **19**

was added for a reaction concentration of 10 µM (1:1 ratio of probe **23**: compound **19**), 50 µM (1:5), 150 µM (1:15) and 500 µM (1:50), respectively, and incubated for 1 h at room temperature in the dark. A 1 mM stock solution of probe **23** was added to all samples except negative and minimal aromatic crosslinker controls to give a 10 µM reaction concentration and incubated in the dark at room temperature for 1 h. To profile unspecific binding to photo-crosslinkers, the minimal aromatic photo-crosslinker **24**[6] was added to the corresponding samples for a reaction concentration of 10 µM. For negative controls, the addition of probes was omitted, but the incubation period still observed. After incubation, the photoreactive diazirine moiety was activated to link covalently to the probe's binding partners by irradiation with 364 nm UV light for 10 min while the samples were placed on cooling packs.

7.3 Click reaction

In order to click the respective probe to the fluorescent rhodamine via the alkyne handle, 4.8 µL of a master mix containing 625 µM rhodamine azide, 12.5 mM $CuSO_4$ (Roth), 835 µM Tris((1-benzyl-4-triazolyl)methyl)amine (TBTA; TCl), and 12.5 mM tris(2-carboxyethyl)phosphine (TCEP; Sigma-Aldrich) was added to each sample. The click reaction mix was allowed to incubate in the dark for 90 min while rotating at 950 rpm.

7.4 SDS-PAGE

The reaction was quenched by mixing the samples 1:1 with 2x Lämmli loading buffer (63 mM Tris-HCl, 10% glycerol, 2% SDS, 0.0025% bromophenol blue, 5% 2-mercaptoethanol). A 4% (w/v) acrylamide (in 50 mM Tris, pH 6.8) gel was used for stacking gels and 12.5% (w/v) acrylamide (in 300 mM Tris, pH 8.8) gel for resolving gels. 35 µL of the mix were loaded per lane. One lane contained a mix of fluorescence (BenchMark, Invitrogen) and protein molecular weight markers (PeqGold Protein Marker I, VWR). The gel was run in SDS buffer (25 mM Tris base, 190 mM glycerol, 0.1% SDS) in the dark at 120 V on a EV265 Consort power supply (Hoefer) before fluorescence imaging.

The fluorescence was scanned in a LAS-4000 imaging system equipped with a Fujinon VRF43LMD3 lens and a 575DF20 filter (Fujifilm).

The loading control was stained with Coomassie dye (0.25% (w/v) Coomassie Brilliant blue R-250, 9.2% acetic acid, 45.4% ethanol in water) overnight and destained for 2 days in an aqueous mix of acetic acid and ethanol in water (1:2:7) with at least two changes of the destaining solution. Absorption of the stained gel was scanned with the same imaging system as the fluorescence.

7.5 Post-processing

All scans (Figures S2 to S7) were cropped and annotated using Gimp version 2.10.36. The images were inverted where necessary for an easier black on white readability. Normalization was performed optically by defining the darkest band as black and the background as white, thus cropping the dynamic range to optimize the visibility of bands. The marker bands (M) were annotated with the corresponding weight in kDa.

7.6 Complete SDS-PAGE scans

Figures S2-S7 show the entire gel area of the SDS-PAGE scans used for Figures 4a and b. Marker lines (M) are annotated with the corresponding molecular weight in kDa.

Equally, the lanes are annotated with the protein samples used for the experiment as well as the respective probe and competitor.

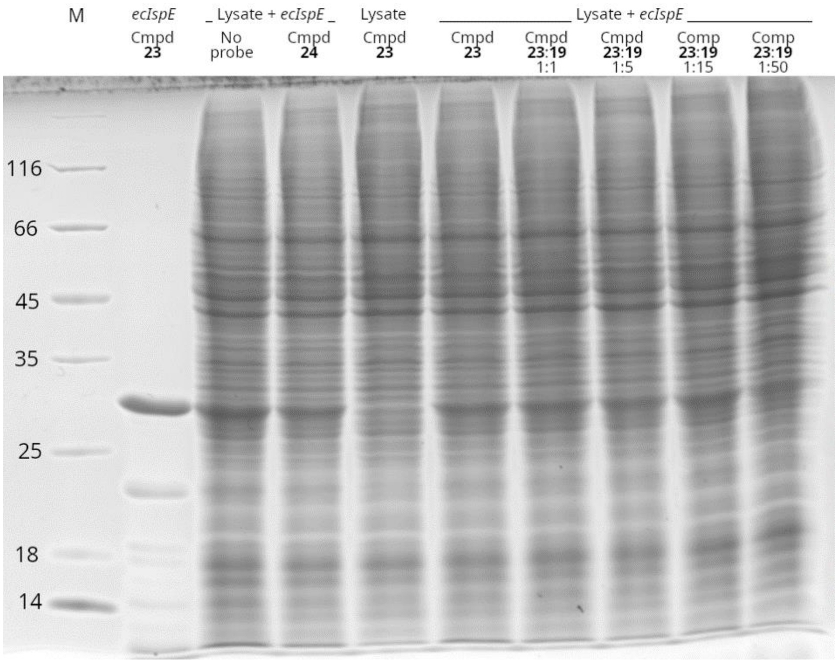

Figure S2: Scan of Coomassie stained gel. Dark bands indicate the presence of protein. *Ec*IspE (UniProt entry P62615) is expected to have a molecular weight of 31 kDa.

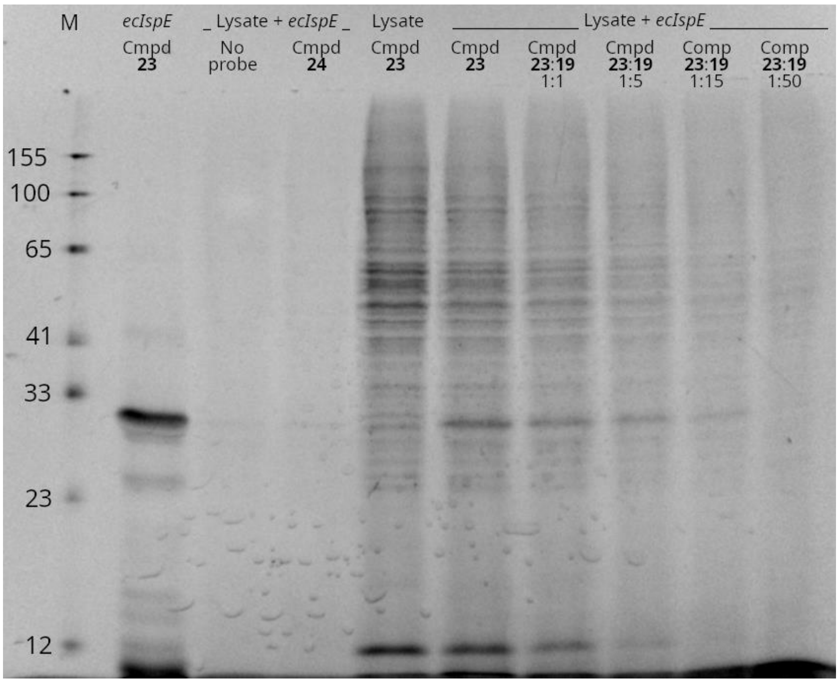

Figure S3: Fluorescence scan of the gel in Figure S2. Dark bands indicate the presence of the fluorescent Rhodamine dye that was clicked to the respective probe, which in turn was covalently linked to proteins in close proximity.

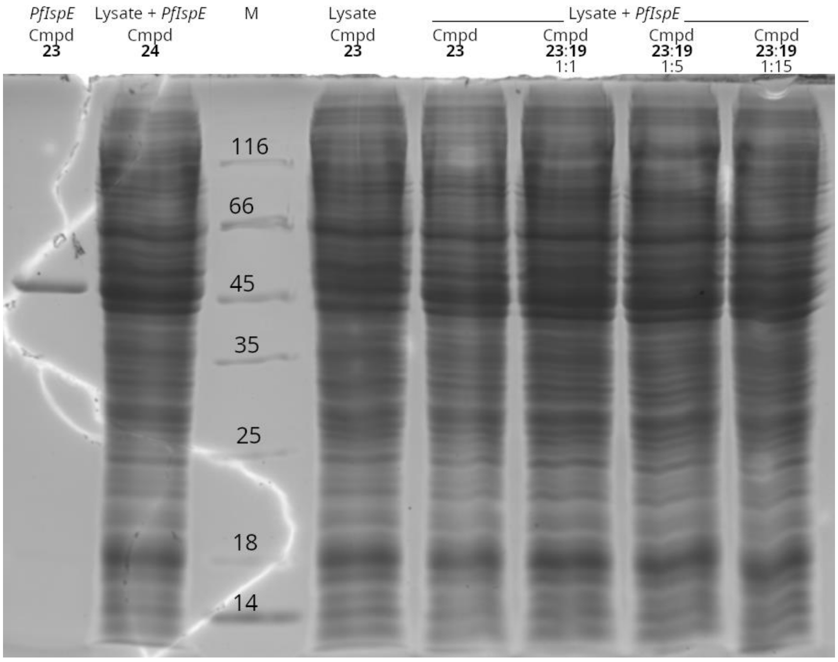

Figure S4: Scan of Coomassie stained gel.
*Pf*IspE (UniProt entry A0A1B1TK84) is expected to run at a molecular weight of 63 kDa.

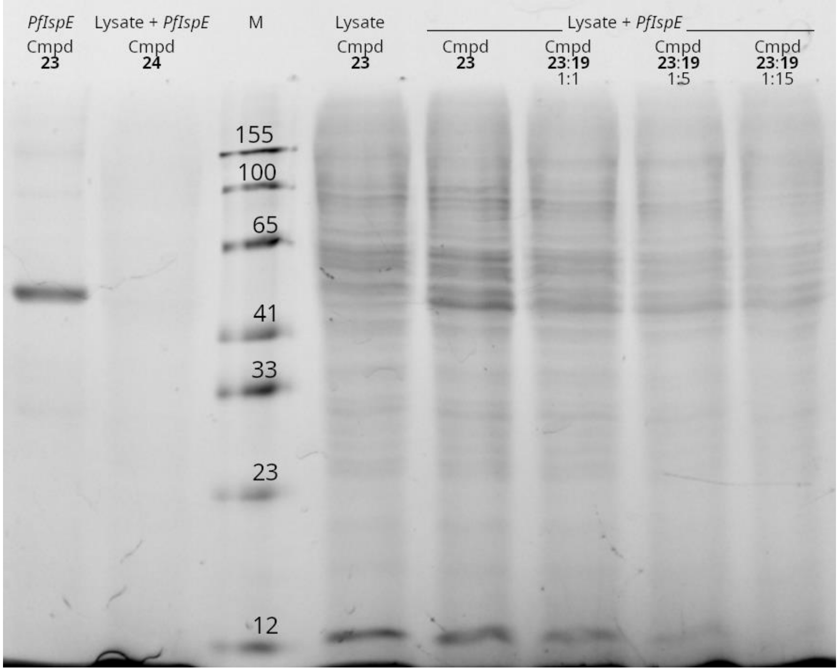

Figure S5. Fluorescence scan of the gel in Figure S4.

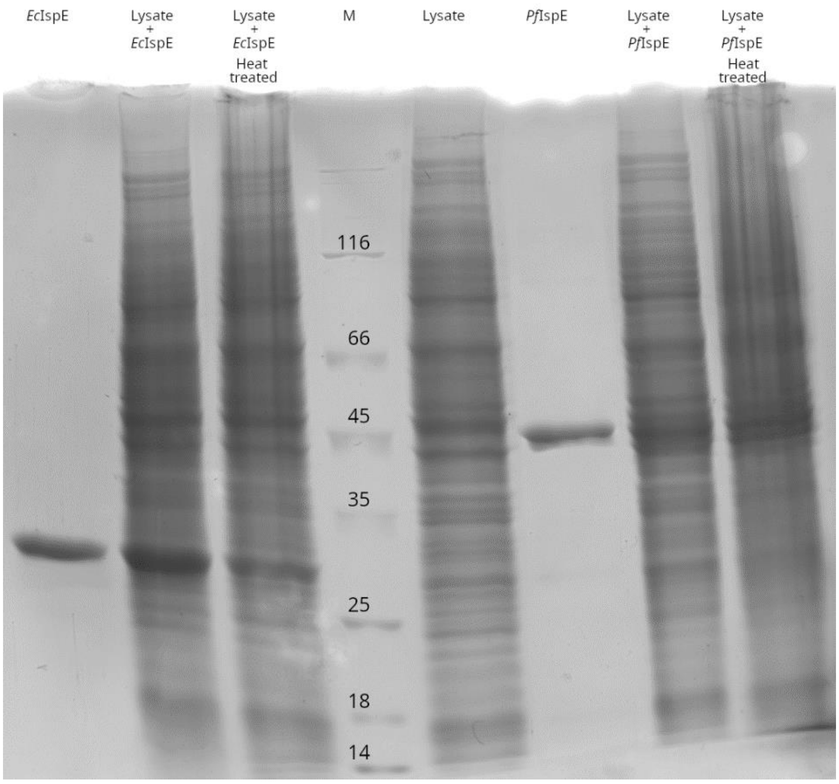

Figure S6. Scan of Coomassie stained gel.

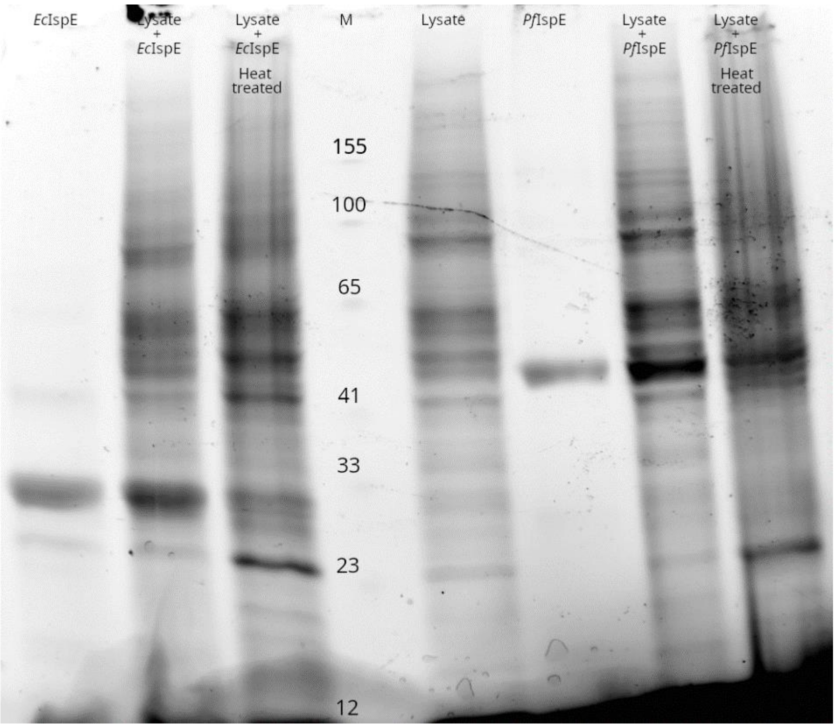

Figure S7. Fluorescence scan of the gel in Figure S5. All samples in this experiment were incubated with probe **23**. Heat treatment before the incubation with the probe is expected to change the affinity between probe and protein due to conformation changes.

7.7 Quantification of relative fluorescence in competition experiments

In order to quantify the brightness of the bands in the competition experiment (Figures S2-S5), we employed the Python-based IOCBIO-Gel program.[7]

The area around the respective IspE bands was selected on both Coomassie stained gel images and fluorescence images after selecting the resolving gel as the region of interest, subtracting a linear background and defining the lanes. The program then calculates the intensities of the areas of interest and converts them additionally into normalized values relative to a reference lane, in this case the lane with compound **23**, but without added competitor compound **19**. The ratio of the normalized fluorescence values divided by the normalized Coomassie stain values are plotted in Figure S8, as a measure of the competitor's effect on probe binding.

The presence of the competitor in 15 times excess results in a reduction in fluorescence by 63% for *Ec*IspE and 48% for *Pf*IspE, respectively.

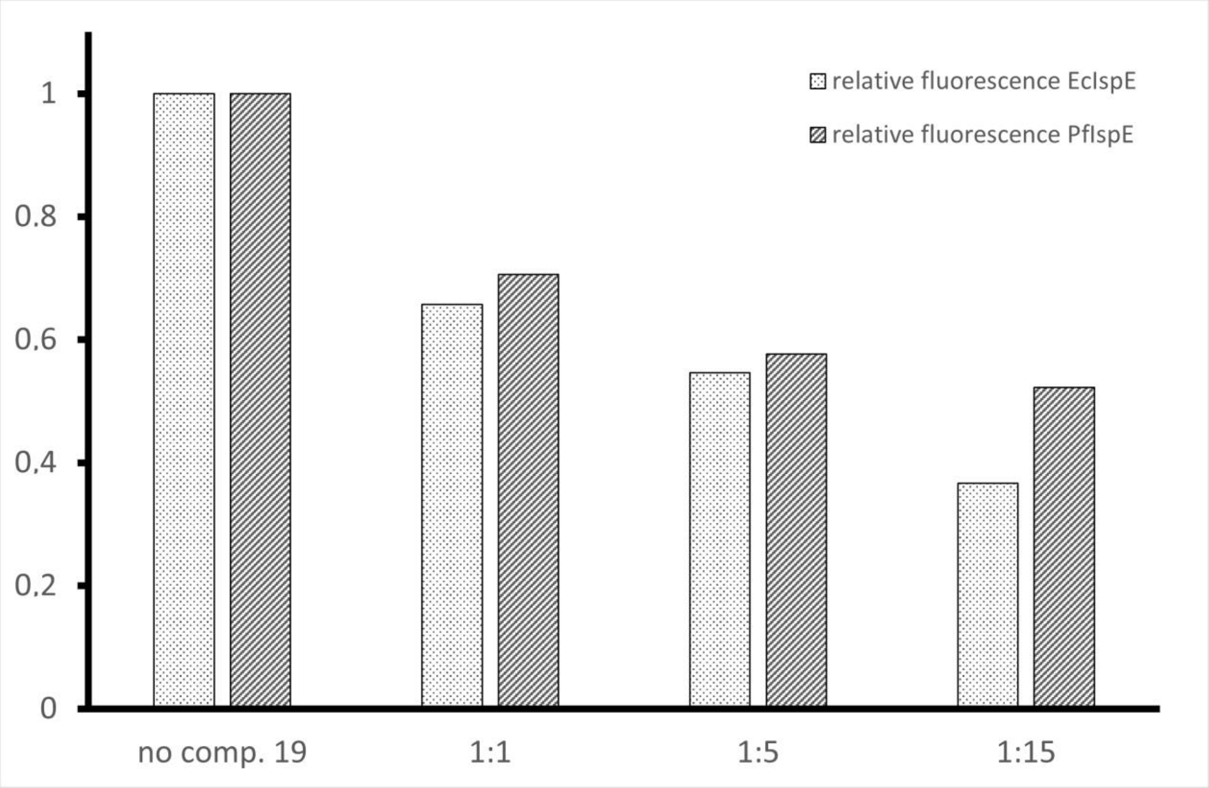

Figure S8. Relative normalized fluorescence in competition series with EcIspE and PfIspE. All samples in this experiment were incubated with probe **23** and reacted with a fluorescent reporter. Additionally, the samples were allowed to react with increasing amounts of the competing active compound **19**. Given here are the ratios of probe **23** to compound **19**. The resulting fluorescence and protein loading was

imaged and quantified. The subsequent values were normalized and plotted as relative fluorescence values to protein loading.

Spectra of some representative compounds

Figure S9: ^{1}H-NMR, ^{13}C-NMR and HPLC-MS of compound **16**

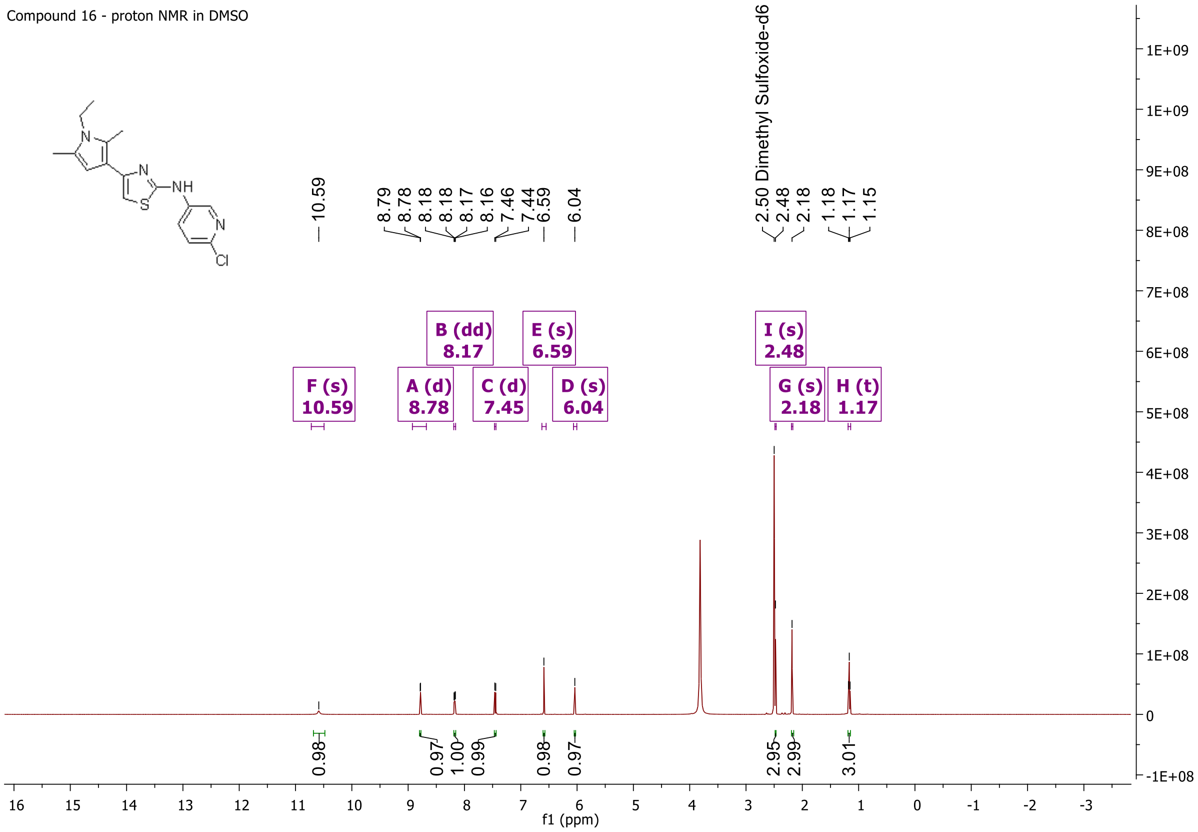

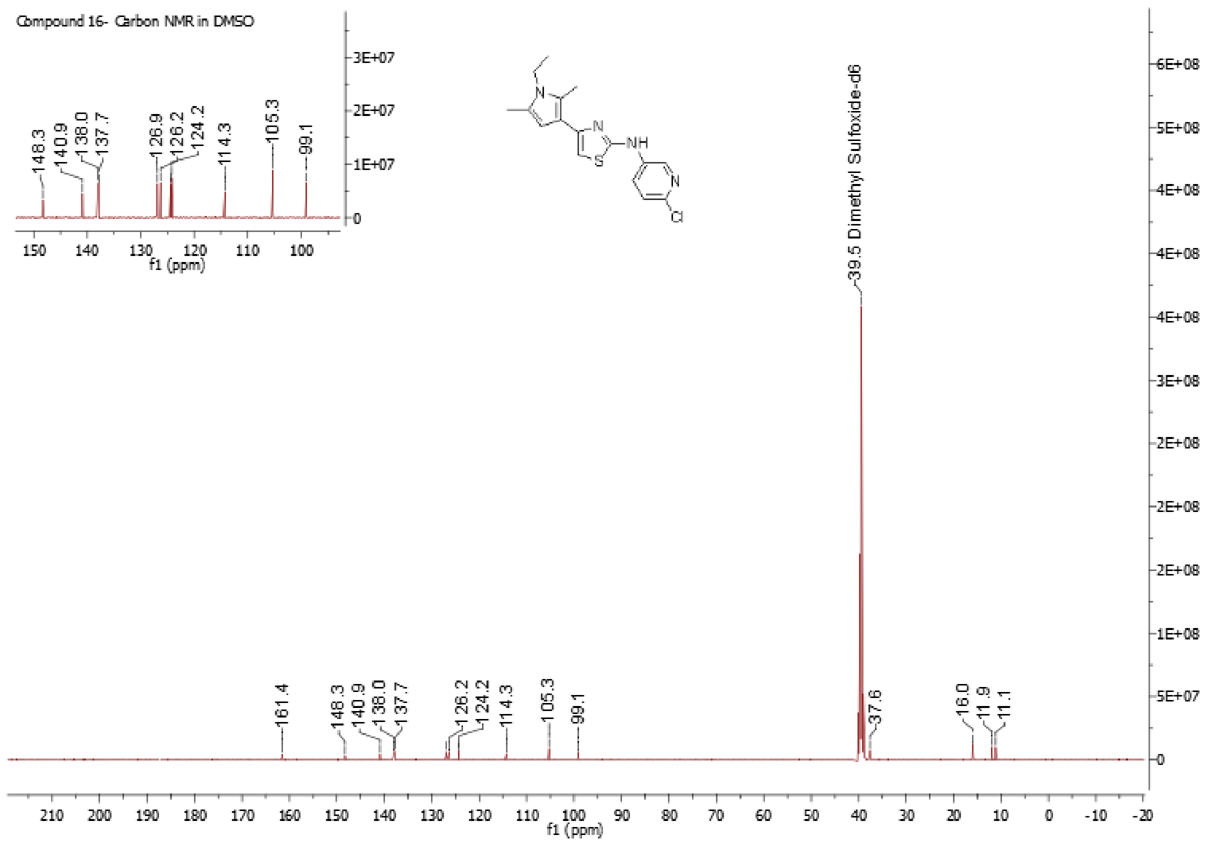
Compound 16- Carbon NMR in DMSO
148.3
140.9
138.0
137.7
126.9
126.2
124.2
114.3
105.3
99.1
f1 (ppm)
161.4
126.2
39.5 Dimethyl Sulfoxide-d6
37.6
16.0
11.9
11.1
NH
N
S
Cl

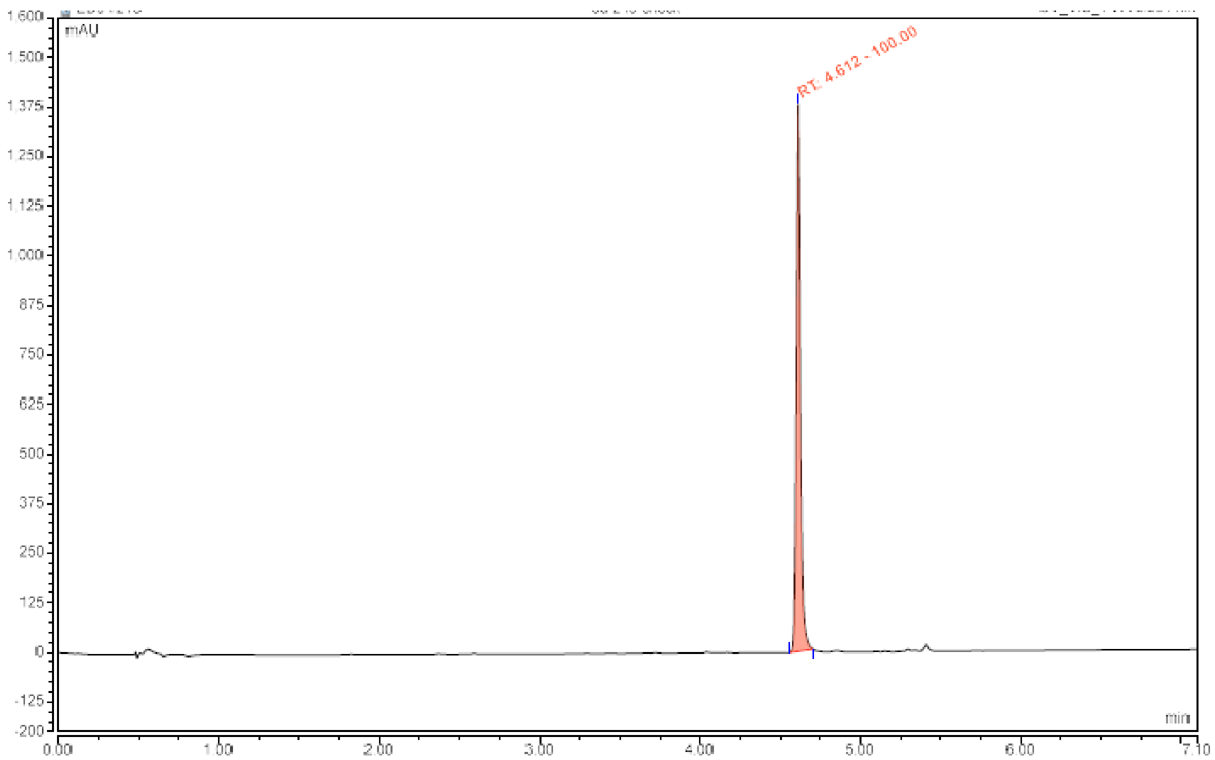
mAU
RT: 4.612 - 100.00
min

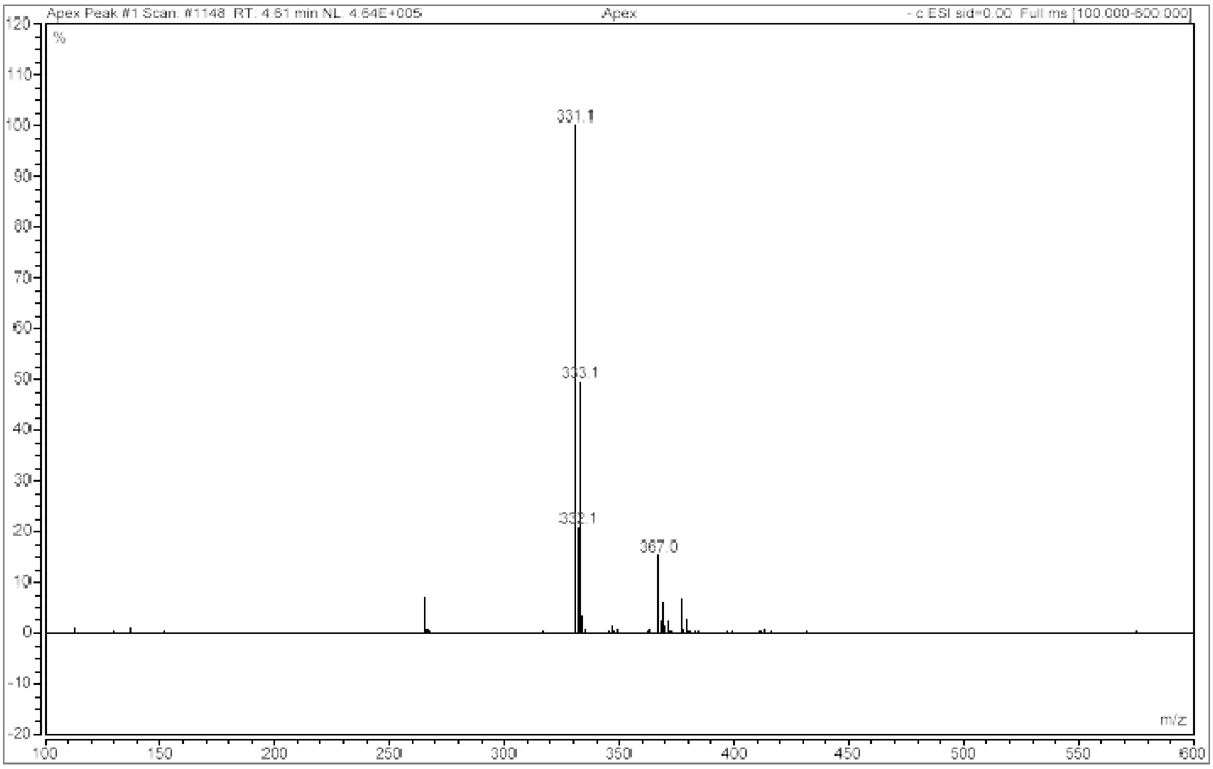

Figure S10: ^{1}H-NMR, ^{13}C-NMR and HPLC-MS of compound **19**

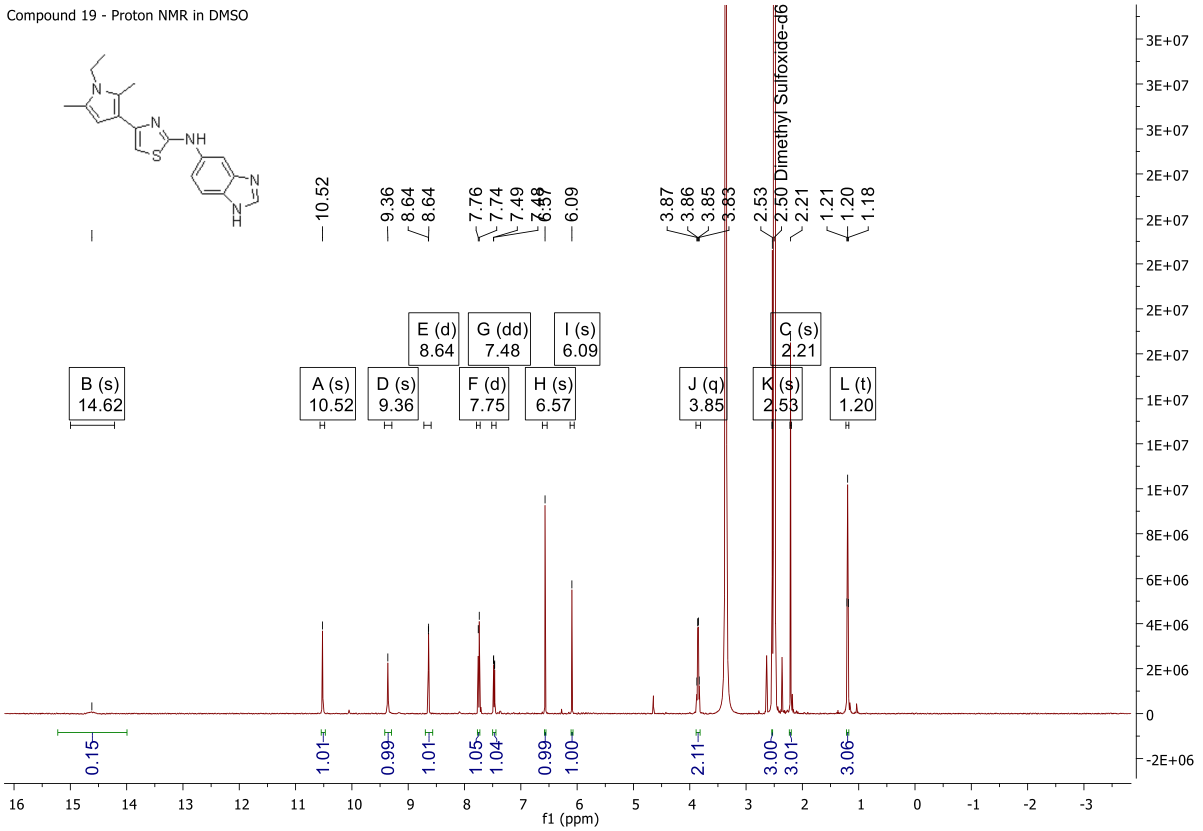

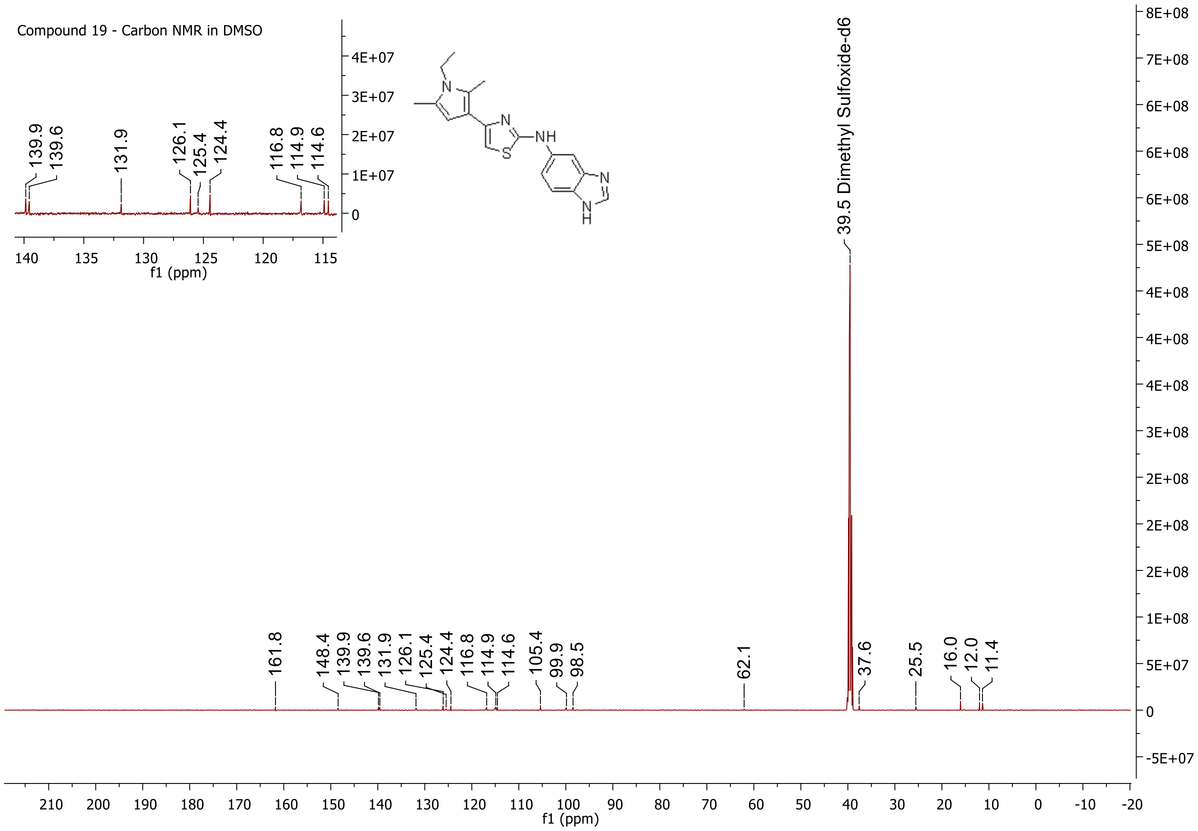
Compound 19 - Carbon NMR in DMSO
39.5 Dimethyl Sulfoxide-d6
161.8
148.4
139.9
139.6
131.9
126.1
125.4
124.4
116.8
114.9
114.6
105.4
99.9
98.5
62.1
37.6
25.5
16.0
12.0
11.4
f1 (ppm)

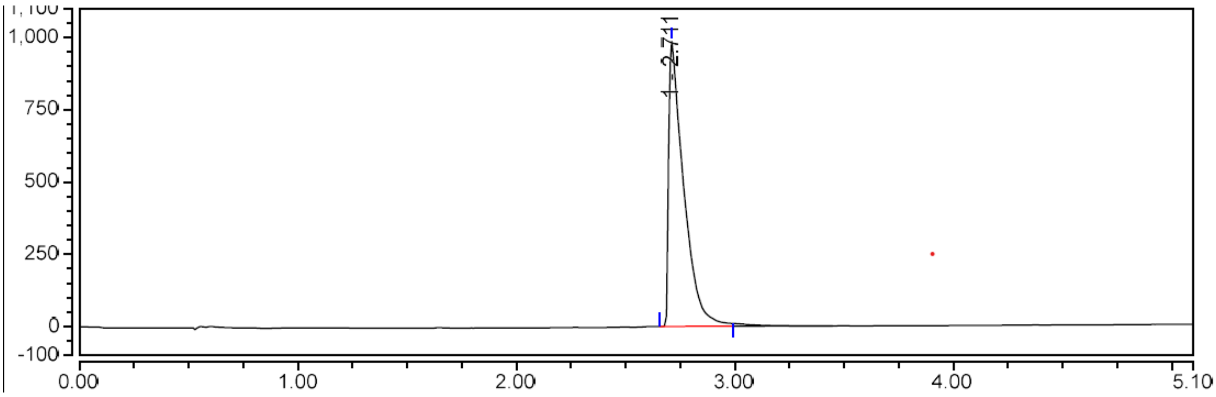
2.71

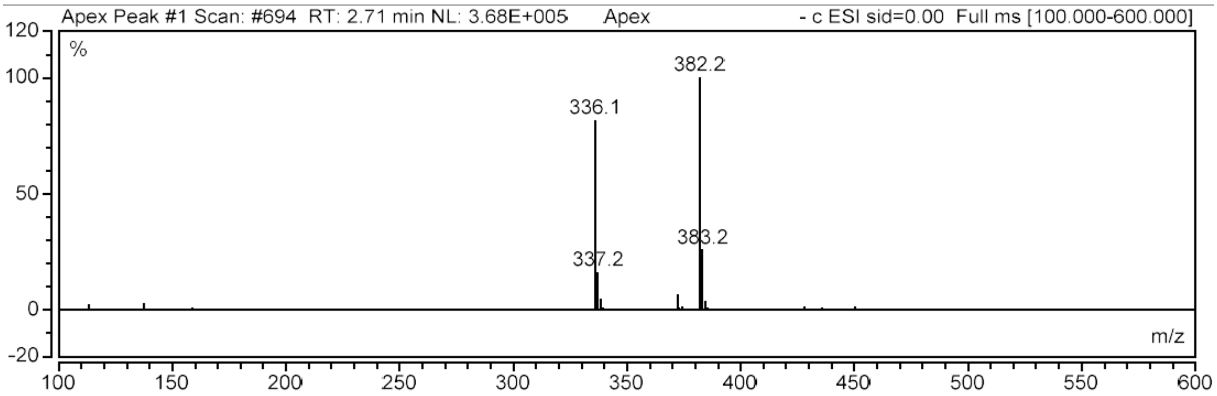
Apex Peak #1 Scan: #694 RT: 2.71 min NL: 3.68E+005 Apex - c ESI sid=0.00 Full ms [100.000-600.000]
%
382.2
336.1
383.2
337.2
m/z

Figure S11: ^{1}H-NMR, ^{13}C-NMR, ^{19}F-NMR and HPLC-MS of **22**

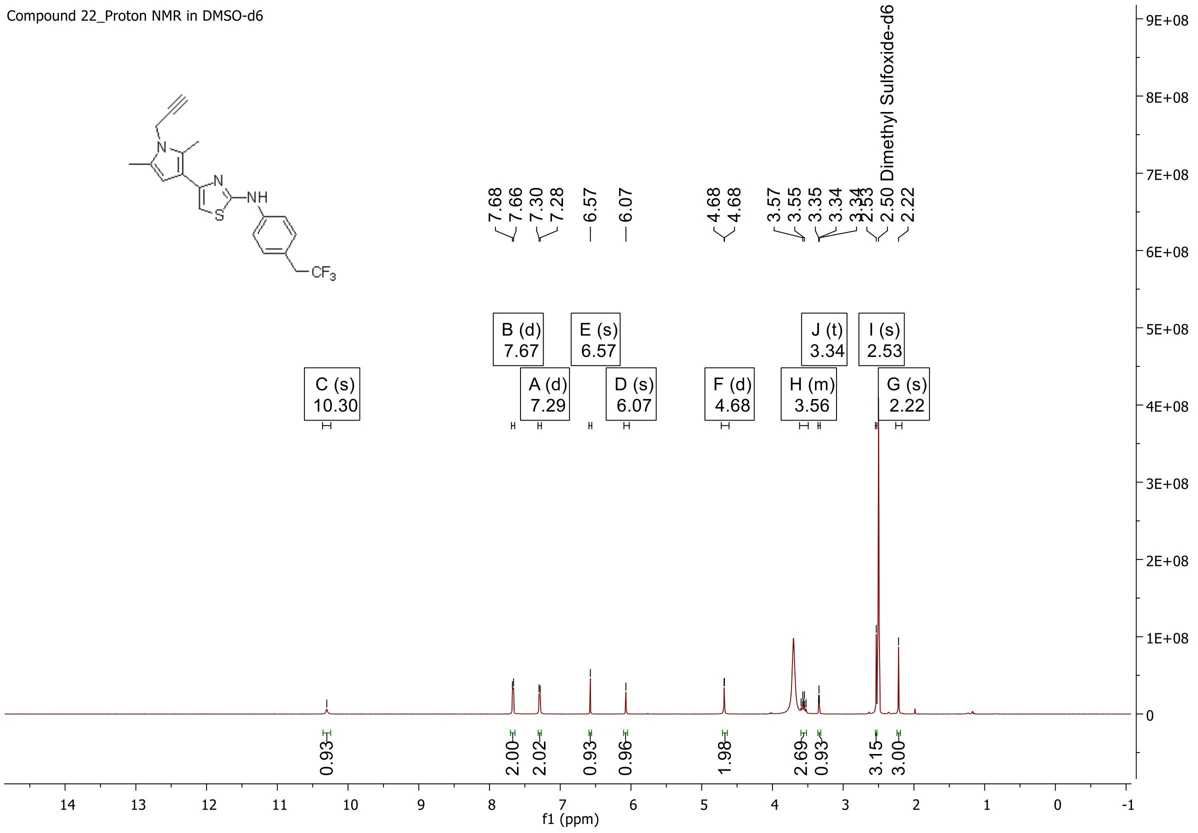

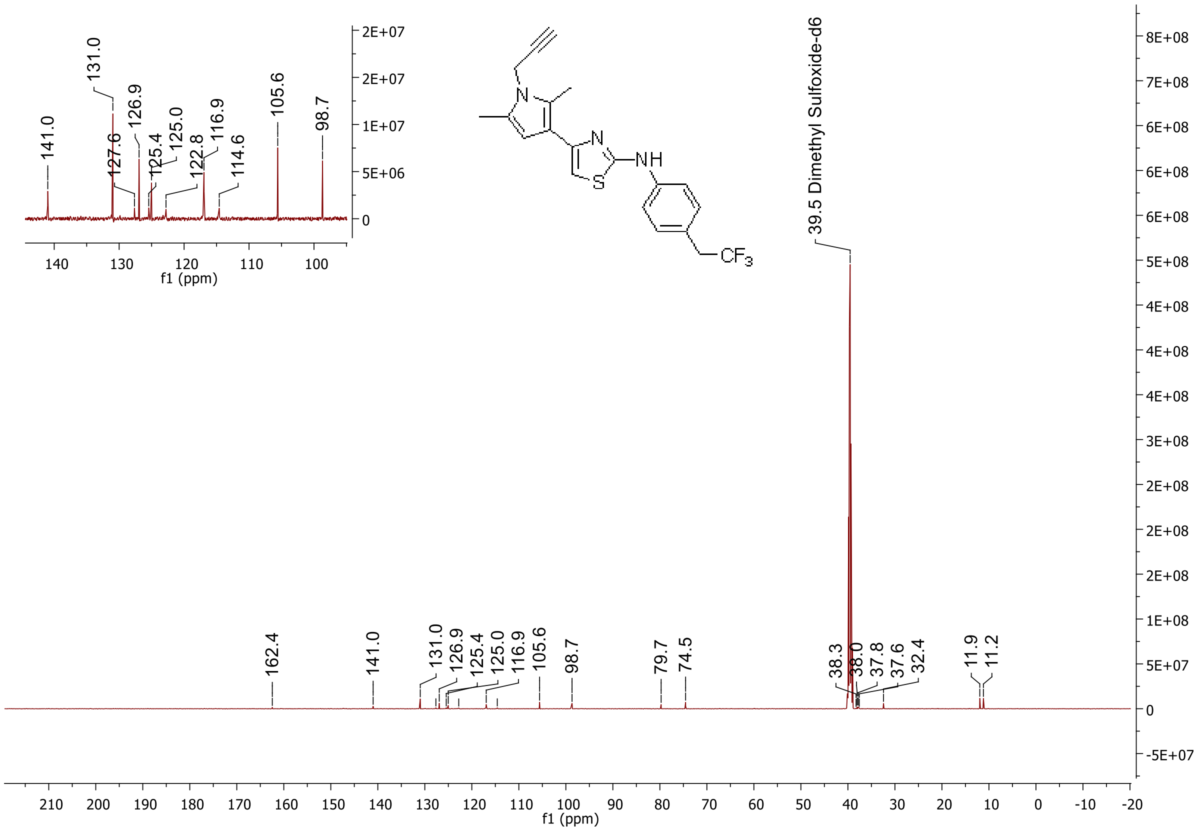
141.0
131.0
127.6
126.9
125.4
125.0
122.8
116.9
114.6
105.6
98.7
f1 (ppm)
N
N
NH
S
CF3
39.5 Dimethyl Sulfoxide-d6
162.4
141.0
131.0
126.9
125.4
125.0
116.9
105.6
98.7
79.7
74.5
38.3
38.0
37.8
37.6
32.4
11.9
11.2
f1 (ppm)

Compound 22

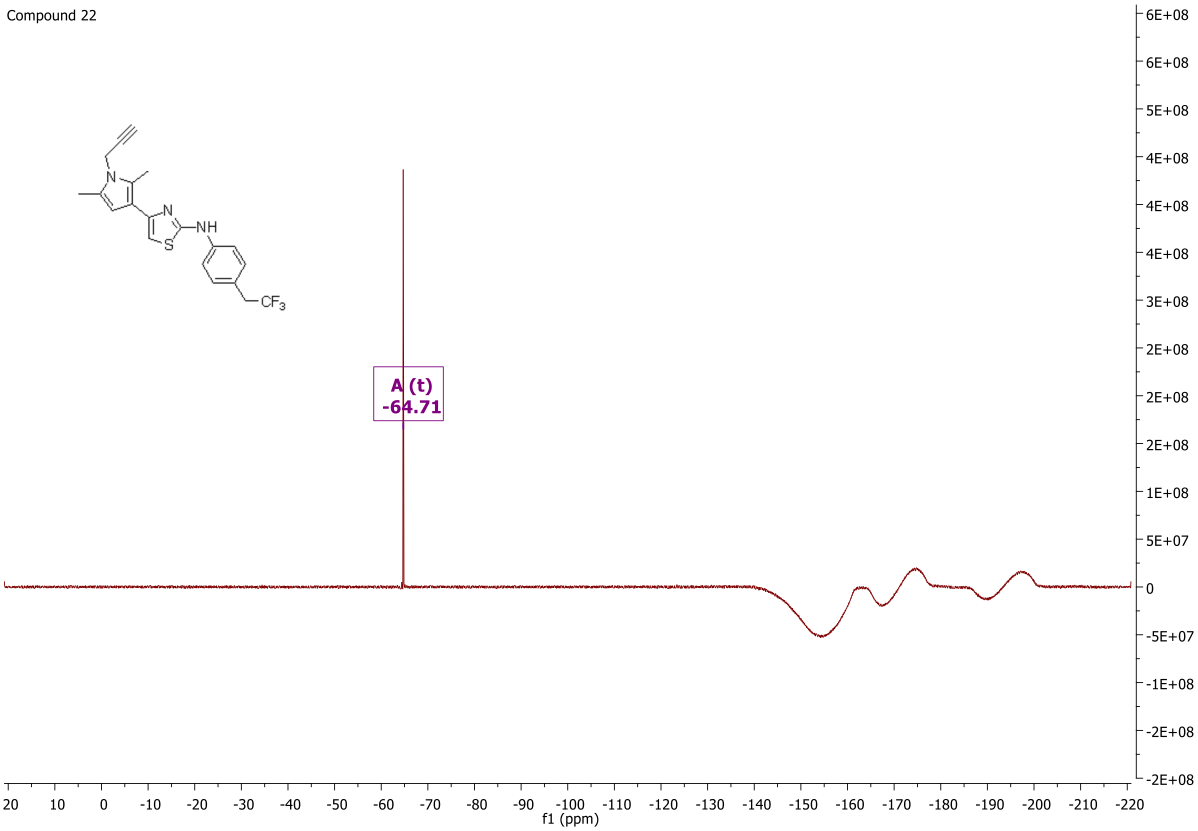
N
N
NH
S
CF3
A (t)
-64.71
f1 (ppm)

Figure S12: ^{1}H-NMR, ^{13}C-NMR, ^{19}F-NMR and HPLC-MS of compound **23**

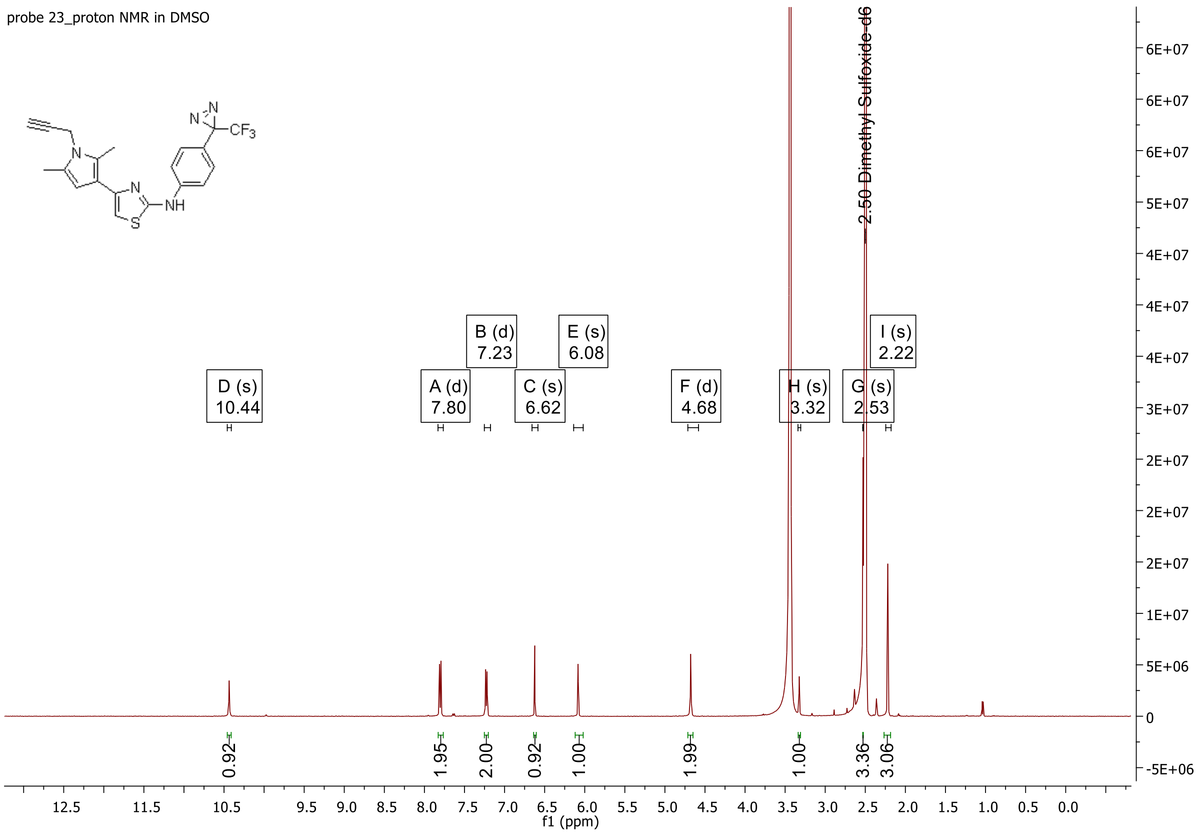

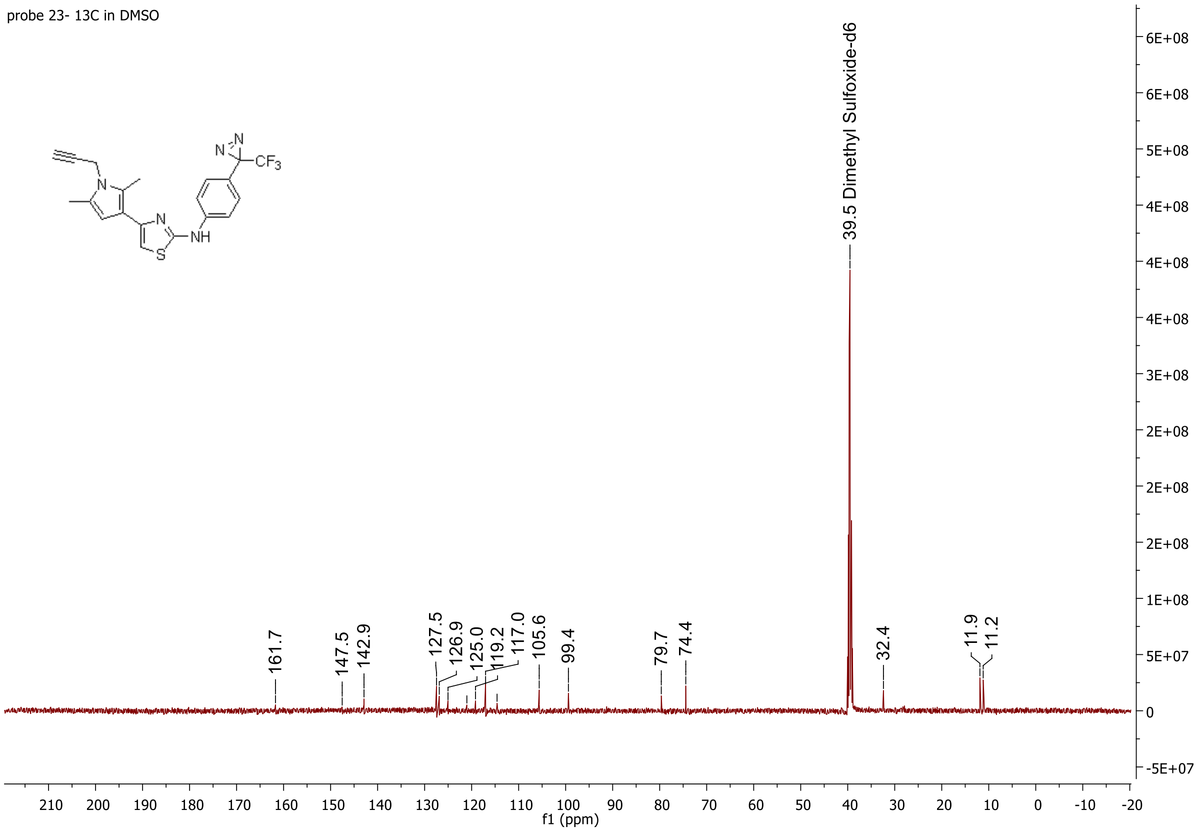

probe 23

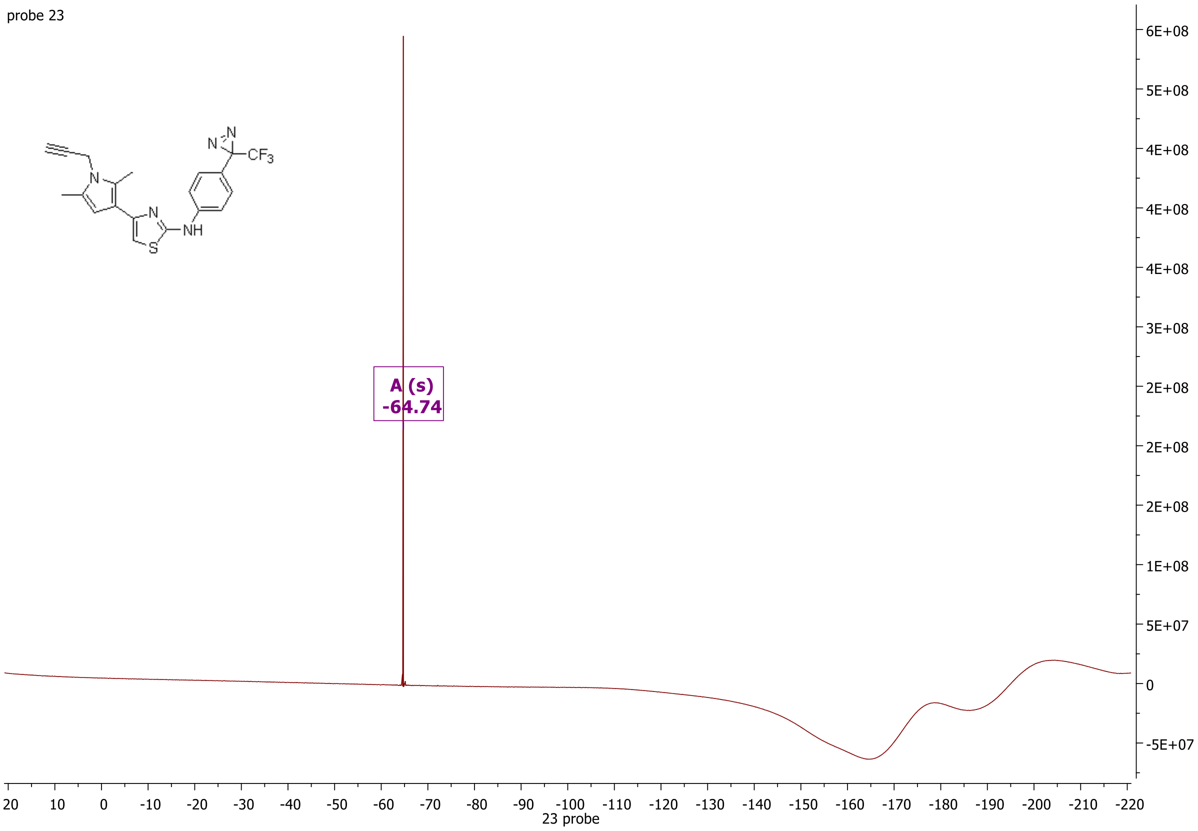

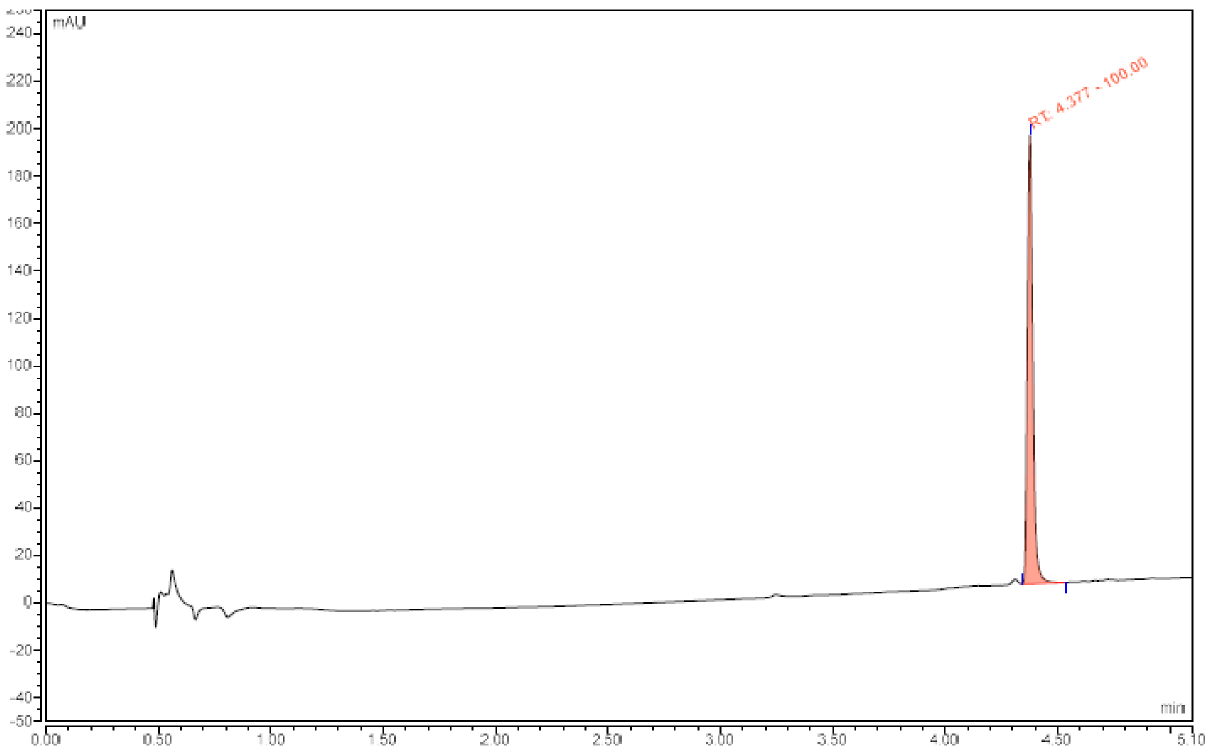

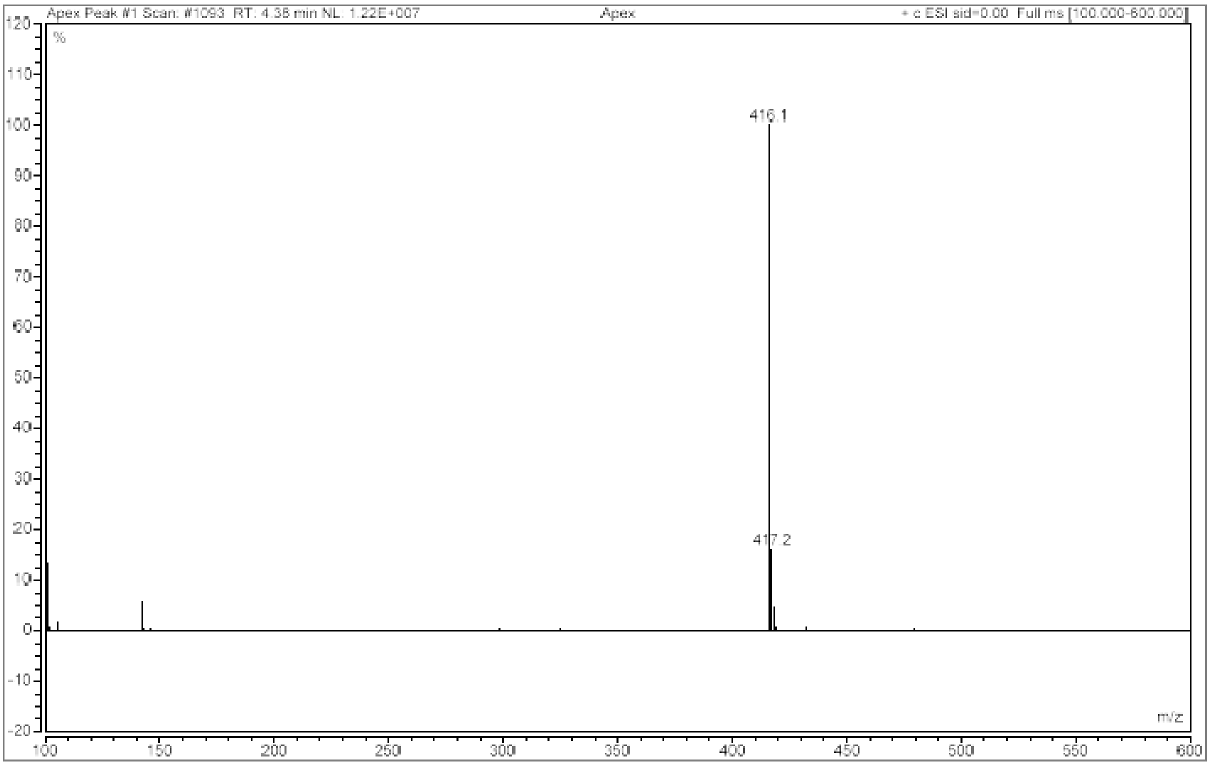

References

[1] Illarionova, V.; Kaiser, J.; Ostrozhenkova, E.; Bacher, A.; Fischer, M.; Eisenreich, W.; Rohdich, F. Nonmevalonate Terpene Biosynthesis Enzymes as Antiinfective Drug Targets: Substrate Synthesis and High-Throughput Screening Methods *J Org Chem* **2006**, *71*, 8824-8834. doi.org/10.1021/jo061466o.

[2] Ropponen, H. K.; Diamanti, E.; Siemens, A.; Illarionov, B.; Haupenthal, J.; Fischer, M.; Rottmann, M.; Witschel, M.; Hirsch, A. K. H. Assessment of the rules related to gaining activity against Gram-negative bacteria *RSC Med Chem* **2021**, *12*, 593-601. doi.org/10.1039/D0MD00409J

[3] Kuzmic, P. Program DYNAFIT for the analysis of enzyme kinetic data: application to HIV proteinase *Anal Biochem* **1996**, *237*, 260-273. doi: 10.1006/abio.1996.0238.

[4] Snyder, C.; Chollet, J.; Santo-Tomas, J.; Scheurer, C.; Wittlin, S. In vitro and in vivo interaction of synthetic peroxide RBx11160 (OZ277) with piperaquine in Plasmodium models *Exp Parasitol* **2007**, *115*, 296-300. DOI: 10.1016/j.exppara.2006.09.016.

[5] Huber, W. ; Koella, J. C. A comparison of three methods of estimating EC50 in studies of drug resistance of malaria parasites. *Acta Trop* **1993**, *55*, 257-261. doi.org/10.1016/0001-706X(93)90083-N

[6] Kleiner, P.; Heydenreuter, W.; Stahl, M.; Korotkov, V.S.; Sieber, S.A. A Whole Proteome Inventory of Background Photocrosslinker Binding *Angew. Chem. Int. Ed.* **2017**, *56*, 1396-1401. doi.org/10.1002/anie.201605993.

[7] Kütt, J.; Margus, G.; Kask, L.; Rätsepso, T.; Soodla, K.; Bernasconi, R.; Birkedal, R.; Järv, P.; Laasmaa, M.; Vendelin, M. Simple analysis of gel images with IOCBIO Gel. *BMC Biol* 21, **2023**, *225* doi.org/10.1186/s12915-023-01734-8.
